# Boosting salinity resilience and silymarin production in *Silybum marianum*: a sustainable strategy using mango-residue biochar and foliar α-tocopherol

**DOI:** 10.1186/s12870-026-08900-4

**Published:** 2026-05-13

**Authors:** Samah M. Youssef, Ibrahim A. A. Mohamed, Yosra S. H. Rateb, Mahmoud A. Hassanain, Marwa Elsaid Hassan, Ahmed Ismail

**Affiliations:** 1 Horticulture Department, Faculty of Agriculture, Fayoum University, Fayoum, 63514 Egypt; 2 Botany Department, Faculty of Agriculture, Fayoum University, 63514 Fayoum, Egypt; 3 Herbal pharmaceutical product analysis administration, Herbal product analysis lab, Egyptian Drug authority, Giza, Egypt; 4 Pharmacognosy Department, Faculty of Pharmacy, Fayoum University, Fayoum, 63514 Egypt

**Keywords:** Biochar, Milk thistle, Osmotic adjustment, Salinity stress, Silymarin, α-tocopherol, Yield

## Abstract

**Background:**

Soil salinity (ECe=8.55 dS m-1), a predominant abiotic stress factor, significantly impairs the growth, yield and pharmaceutical quality of the medicinal plant *Silybum marianum* (milk thistle).

**Objective:**

This study evaluated the potential of mango-residue biochar and foliar α-tocopherol (α-Toco) on the physiological stability, agronomic performance, and pharmaceutical quality of milk thistle under saline conditions.

**Methods:**

A two-season field trial was performed using a split-plot arrangement of biochar amendments (0–15 t ha⁻¹) and α-Toco applications (0–150 ppm).

**Results:**

The integrated application of two treatments effectively mitigated salinity stress, leading to significant increases in growth and productivity. The optimal treatment (10 t ha⁻¹ of biochar and 150 ppm α-Toco) markedly enhanced both above- and below-ground biomass compared to the control. This synergistic effect was attributed to improved nutrient uptake (such as N, P, K^⁺^, Mg^2⁺^, Zn^2⁺^), and reduced Na^+^ accumulation. Furthermore, the highest treatment combination (15 t ha⁻¹ biochar × 150 ppm α-Toco), dramatically enhanced the antioxidant system by increasing endogenous α-tocopherol and ascorbate peroxidase activity. This treatment effectively alleviated oxidative damage, as evidenced by significant reductions in proline, H_₂_O_₂_ levels, and IC₅₀ values. Most importantly, these physiological improvements resulted in the highest seed yield (1.95 t ha⁻¹) and a significant increase in silymarin content (up to 3.172%).

**Conclusions:**

The synergy between biochar and α-Toco provides a sustainable strategy for enhancing milk thistle resilience and pharmaceutical quality in saline environments, offering a promising approach for the productive utilization of marginal lands under climate change conditions.

**Graphical Abstract:**

**Figure:**
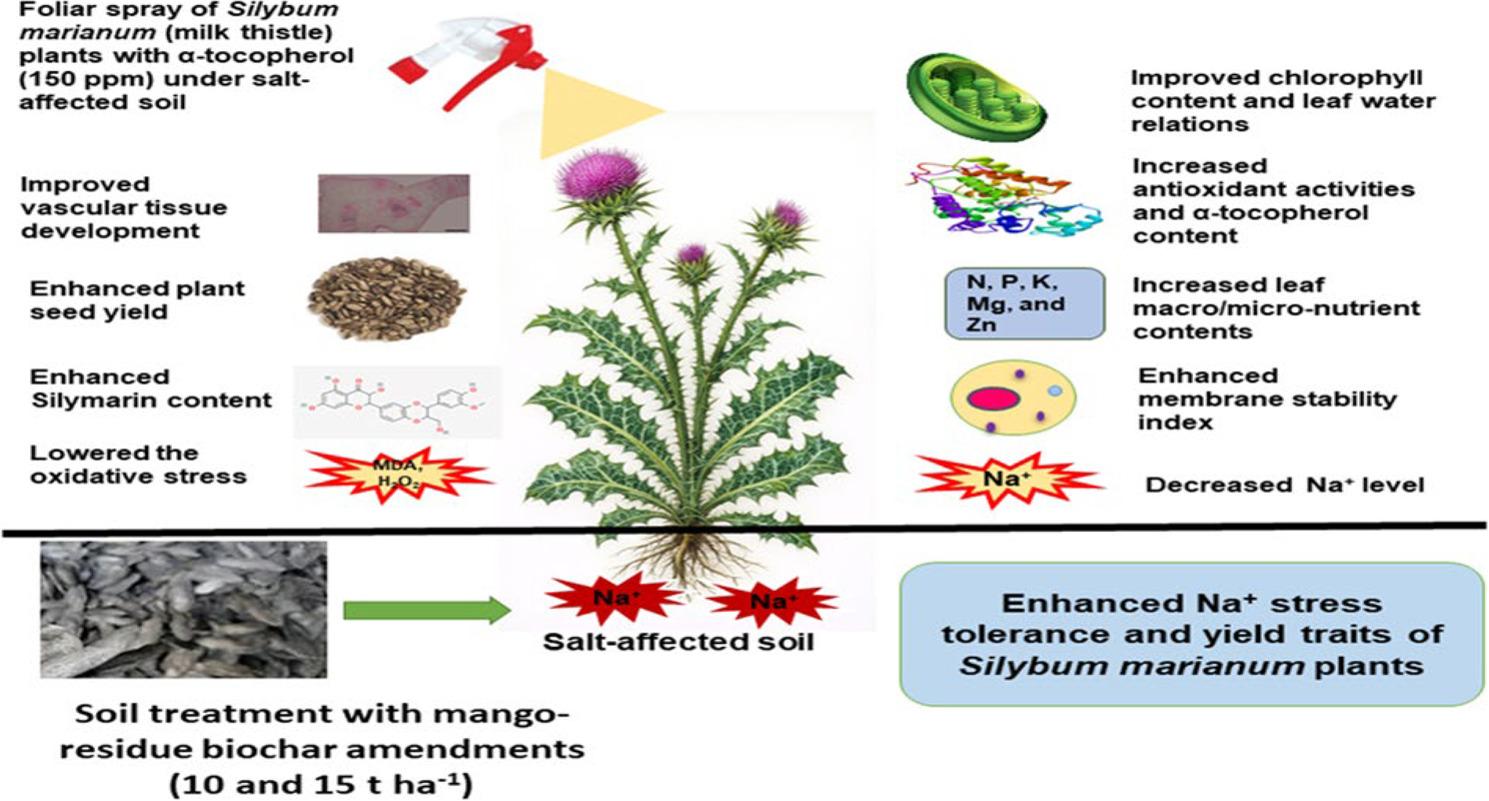
A dual role for biochar and α-tocopherol: Unveiling the protective pathways while stimulating growth and productivity

## Introduction

**Fig. 1:**
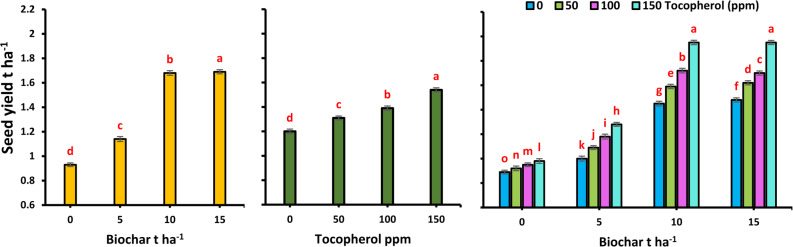
Effect of biochar application and α-tocopherol, and their interaction on seed yield t ha^− 1^ of milk thistle grown in (S_I_) 2023/24 and (S_II_) 2024/25 winter seasons. Results are presented as means ± standard error (*n* = 3). Mean values in the same bar followed by a distinct lowercase letter are significantly different according to Tukey’s HSD test (*p* ≤ 0.05)

Climate change is a serious threat to agriculture and food supplies in the 21st century [1]. One major way this threat plays out is through increased soil salinization, largely caused by climate-related processes, particularly in dry and semi-arid regions [2, 3]. Globally, over 833 million hectares of arable land are affected by salinity, resulting in estimated annual crop losses exceeding $12 billion [4, 5]. In soil substrates, high salt levels cause harmful effects from sodium ions and also make it harder for plants to access important nutrients like nitrogen, phosphorus, potassium, calcium, iron, manganese, copper, and zinc [6]. This salinity not only reduces the nutrient uptake but also promotes oxidative stress through the production of reactive oxygen species (ROS) [7, 8]. Hydrogen peroxide and superoxide, as well as hydroxyl radicals, also contribute to these acute problems [9]. These ROS target cellular components such as membrane lipids; for instance, they exacerbate lipid peroxidation, destabilizing membranes and disrupting nutrient homeostasis [10]. This scenario is particularly critical in Egypt, where salinity affects 15% of cultivated land, necessitating sustainable soil management to maintain medicinal crop productivity [11, 12]. Strategies to mitigate stress are vital for thriving medicinal seed in these challenging circumstances (Fig. 1).

*Silybum marianum* (L.) Gaertn., commonly referred to as the milk thistle plant, is a highly researchable and economically important plant [13]. This species is a member of the Asteraceae family, and its main quality lies in the harvested silymarin complex —comprised of flavonolignans—that is obtained from its high-quality seed and is famous for its very potent hepatoprotection, antioxidant, and anti-inflammatory activity [14]. Additionally, these bioactivities may be complemented with additional synergistic compounds, such as taxifolin and quercetin glycosides, for significant anti-inflammatory activities [15]. In addition to its richness of compounds, milk thistle has a high intrinsic resistance; in fact, it is known for being a species with a heterozygous genome that shows particular tolerance to different abiotic stresses, such as heat and drought [16]. Although the genetic background of this tolerance to stress and its local adaptation to the Mediterranean habitat are likely, until now, little is known about the physiological and biochemical mechanisms involved in its ability to respond to high salinity conditions. Therefore, the possibility of further improvements in performance that could secure stable yields of silymarin from salt-affected marginal lands is particularly crucial and little explored.

Sustainable soil amendments represent a promising strategy to alleviate the edaphic limitations of salinity. As a sustainable amendment, biochar improves soil physical properties through its large surface area and porous structure, which enhances aeration and water retention [17, 18]. Chemically, biochar may increase cation exchange capacity and nutrient availability while decreasing the bioavailability of Na^+^ ions for plant uptake by adsorption [19]. Several investigations have reported that the use of biochar can alleviate salinity stress of plants and enhance the overall soil and root zone quality [20, 21].

The use of protective additives that are applied exogenously is a direct physiological approach to enhance plant traits besides soil-applied amendments. Exogenous alpha-tocopherol (α-Toco) plays a vital role in scavenging lipid peroxyl radicals [22]. By doing so, it effectively halts the lipid peroxidation chain reaction, protecting both the membrane’s stability and its structural integrity. Most importantly, it also prevents oxidative damage to the photosynthetic apparatus [23]. Significant findings emphasize this protective role, showing that topically applied α-Toco enhances plant vigor and photosynthetic capacity. In the case of plants subjected to abiotic stress, including drought, salinity, and heavy metal toxicity, it aids in the improvement of their inherent defense system [24, 25]. From an agronomic perspective, it is essential to address the practical translational feasibility of utilizing α-Toco in field conditions. While the direct application of pure analytical-grade α-Toco is not economically viable as a standalone farm input, it serves as a crucial mechanistic probe in scientific research. In practical agriculture, the stress-mitigating benefits of α-Toco are readily accessed by farmers through commercially registered biostimulants, such as vitamin-enriched amino acid formulations and seaweed extracts (e.g., *Macrocystis pyrifera*), which naturally contain active tocopherol complexes. Therefore, elucidating the precise physiological mechanisms of pure α-Toco—as demonstrated in the present study—provides the essential scientific foundation explaining the mode of action of these widely adopted commercial products. Furthermore, the practical adoption of such biostimulant strategies is highly justified for high-value medicinal crops like milk thistle. The low effective concentrations required for foliar application, coupled with the resultant significant enhancement in silymarin biosynthesis, ensure a highly favorable return on investment. Ultimately, these findings not only validate current commercial biostimulants but also lay the groundwork for developing advanced, cost-effective delivery systems, such as biochar-vitamin composites, specifically tailored for reclaiming marginal saline lands.

Although previous research has independently documented the benefits of biochar for soil conditioning and α-Toco for stress alleviation, their combined efficacy in medicinal plants under salinity stress remains largely unexplored. We hypothesized that the co-application of mango-residue biochar and foliar α-Toco acts as a dual-defense strategy: biochar improves soil physicochemical properties and restricts sodium uptake, while α-Toco stabilizes cellular membranes and reinforces the antioxidant system. The novelty of this work lies in its pioneering field-based evaluation of this synergistic approach in milk thistle, offering a comprehensive analysis that bridges soil amendment with anatomical modifications, physiological resilience, and pharmaceutical quality (silymarin content). Accordingly, this study was designed to investigate the individual and interactive effects of these treatments on:

- Plant growth and nutrient homeostasis (N, P, K⁺, Mg²⁺, Zn²⁺ and Na^+^).
- Oxidative stress markers and antioxidant defense systems under saline conditions.
- Anatomical modifications, yield components and pharmaceutical quality (silymarin content).

## Materials and methods

### Site and soil attributes

The field experiment was conducted at the Agriculture College Research Farm, Fayoum University, in Fayoum City, Egypt. Nestled in Egypt’s arid climate zone region northwest of the Nile River, it is located at approximately 29° 16’ N latitude and 30° 38’ E longitude. This site is distinguished by its saline sandy loam soil. Based on the salinity classification described by Dahnke and Whitney [26], the experimental soil (ECe = 8.55 ± 0.1 dS m⁻¹) is categorized as strongly saline. Soil samples were gathered from the topsoil layer (0–25 cm depth), allowing us to examine the physical and chemical properties of the soil prior to treatment. After the harvest, we executed a careful examination of soil samples from the most impactful and recommended treatment. Every analytical procedure adhered rigorously to the established standards posed by Klute and Dirksen [27] and Page et al. [28]. Comprehensive findings are available in Tables 1 and 2. The meteorological conditions at the experimental site during the 2023/24 and 2024/25 growing seasons were characteristic of an arid climate. The average maximum and minimum air temperatures ranged from 24.1 °C to 25.4 °C and 11.8 °C to 13.5 °C, respectively. The mean relative humidity fluctuated between 52.3% and 55.6%. Seasonal rainfall was negligible (< 16 mm), necessitating that the crop water requirements be met entirely through irrigation. These historical weather data were obtained from the Visual Crossing weather database (https://www.visualcrossing.com/).

**Table 1:** Initial physical characteristics of the experimental soil

Sand %	Silt %	Clay %	Texture	Ρb gcm^− 3^	K_sat_ Cm h^− 1^	F.C %	W.*P*. %	A.W. %
70.90	15.60	13.50	Sandy Loam	1.53	1.78	22.13	10.45	11.68

*Pb* soil dry bulk density, *K*_*sat*_ soil saturated hydraulic conductivity, *F.C* soil field capacity, *W.P.* soil wilting permanent point, and *A.W.* soil available water. Soil dry bulk density (pb), soil saturated hydraulic conductivity (K_sat_), soil field capacity (F.C), soil wilting permanent point (W.P.), and soil available water (A.W.)

**Table 2:** Soil chemical attributes at the experimental site

Properties	Soil (Before biochar application)	Soil (after biochar application)
pH [at a soil: water(w/v) ratio of 1:2.5]	7.32	7.41
ECe (dS.m^− 1^; soil paste extract)	8.55	6.14
CEC (cmole kg^− 1^)	12.13	14.36
CaCO_3_ (g kg^− 1^)	52.1	53.11
O. M (%)	1.41	1.73
Nutrient content		
*N* total %	0.03	0.05
*P* available (mg kg^− 1^ soil)	5.16	7.64
K available (mg kg^− 1^ soil)	38.98	44.20

*ECe *Electrical conductivity, *CEC* cation exchangeable capacity, *OM* organic matter, *N* nitrogen, *P* phosphorus, *K* potassium, and *CaCO*_3_ calcium carbonate

### Field crop-management and experimental design

Healthly seeds of the local “Purple” milk thistle cultivar were obtained from the Research Center of Medicinal and Aromatic Plants in Giza, Egypt. A field experiment unfolded across two consecutive winter growing seasons: 2023–24 and 2024–25. Seeds were sown immediately on November 1, 2023, and November 3, 2024. We implemented a split-plot design in a randomized complete block arrangement, with three replications for each treatment. Biochar amendments were assigned to the main plots, while α-Toco concentrations were allocated to the sub-plots. Each experimental plot measured a generous 8.4 m² (4 rows × 3.5 m length × 0.6 m width). There were five plants on each ridge for a total of 20 plants per plot. Agronomic operations carried out, including irrigation, cultural practices, and pest and disease control, adhered to the local commercial agricultural standards.

### Preparation of soil amendments and plant growth antioxidant treatments

#### Biochar source and preparation

In this study, the biochar was made from mango tree pruning (*Mangifera indica* L.) from a local carbonization unit in Itsa district, Fayoum Governorate, Egypt. It was manufactured by slow pyrolysis in an oxygen-limited environment within the temperature range of 400–500 °C. After production, the biochar was ground and sieved by 2-mm sieving to homogenize the particle size before application to the soil. The main biochar chemical characteristics was analyzed following the standard protocol described by Page et al. [28] and the outcomes are shown in Table 3.

**Table 3:** Key physical and chemical traits of the biochar amendment

pH [at a soil: water (w/v) ratio of 1:2.5]	ECe (dS.m^− 1^; soil paste extract)	OC %	*N* %	C: *N*	CaCO_3_ %	*P* %	K %
7.31	2.98	59.32	1.97	30.11	2.14	0.26	0.74

*ECe *Electrical conductivity, *OC* organic carbon, *C: N* carbone: nitrogen, *N* nitrogen, *P* phosphorus, *K* potassium, and *CaCO*_*3*_ calcium carbonate

#### Plant growth antioxidant preparation

α-Toco (Sigma-Aldrich, ≥ 95% purity) was initially dissolved in a minimal volume of absolute ethanol (1 mL per 100 mg) to ensure complete solubility. The resulting stock was then diluted with distilled water to obtain the final concentrations (50, 100, and 150 mg L⁻¹), with Tween-20 (0.3 mL L⁻¹) added as a surfactant. The final ethanol concentration in all spray solutions was maintained below 0.15% (v/v) to ensure no phytotoxic effects. Control plants were sprayed with distilled water only.

### Application of treatments

The experiment included two main factors in it: biochar and α-Toco. Biochar amendments were applied at four rates: 0 (control), 5, 10, and 15 t ha⁻¹. α-Toco, a plant growth antioxidant, was applied as foliar sprays at four concentrations: 0 (distilled water control), 50, 100, and 150 ppm. This setup produced a total of 16 factorial treatment combinations. The full biochar dose was thoroughly mixed into the soil two weeks before sowing. At the same time, α-Toco was spray-applied in the field at 30, 45, and 60 days after sowing (DAS) with a handheld sprayer to cover the whole foliage. These timings correlate with the prolonged BBCH stages of *Silybum marianum*, namely BBCH 19 (nine or more fully unfolded leaves), followed by BBCH 31 (beginning of rosette expansion, 10% ground cover) and BBCH 35 (mid-rosette growth, 50% ground cover), respectively [29].

### Growth measurements (BBCH 51; beginning of inflorescence emergence)

Five plants were randomly selected from each replicate and tagged for detailed observation. The following parameters were then measured: plant height, number of branches per plant, above-ground dry biomass per plant (g), root length per plant (cm), and below-ground dry biomass per plant (g).

### Photosynthetic pigment analysis (BBCH 39; complete rosette growth)

Leaf samples for pigment analysis were taken when the plants attained about 90% ground cover. Chlorophyll a (Chl *a*), chlorophyll b (Chl *b*), and total carotenoid (Car) content were analyzed spectrophotometrically from an acetone extract using the Arnon [30] method. Subsequently, the total chlorophyll (total chl) to Car [(Chl *a* + *b*)/Car] ratio was computed.

### Physiological measurements (BBCH 65–67; mid flowering)

Leaf samples were collected and analyzed for some physiological resilience during this peak-demand metabolic phase. Analyses included relative water content (RWC) using the original method devised by Weatherley [31] and later modified by Barrs and Weatherley [32]. The membrane stability index (MSI), calculated using a procedure altered from the original method by Sairam [33], is based on the approach mentioned by Premachandra et al. [34].

### Nutrient analysis (BBCH 65–67; peak flowering)

For measuring nutritional alterations during a highly active period in metabolism, leaf minerals were judged to be the control. Nitrogen (N) was evaluated using colorimetry [35], while phosphorus (P) measured following Jackson’s method [36]. Levels of potassium (K) and sodium (Na) were measured using a Perkin-Elmer model 52 flame photometer [28]. Magnesium (Mg) and zinc (Zn) were conducted using atomic absorption spectrophotometry as applied as described by David [37].

### Leaf phytochemical analysis (BBCH 65–67; peak flowering)

To scrutinize the biochemical processes underlying tolerance, critical stress indicators were evaluated. The amount of total soluble sugar and free proline was determined as the main osmoprotectants following the methods by Irigoyen et al. [38] and Bates et al. [39], respectively. Simultaneously, the assessment of the antioxidant defense system was including total phenolic content (TPC) using Meyers et al. [40], total flavonoid content (TFC) as described by Bahorun et al. [41], and endogenous alpha-tocopherol (EAT) based on Emmerie and Engel [42] techniques. We also determined IC_50_ half-maximal inhibitory levels by means of the 2,2-diphenyl-1-picrylhydrazyl assay, based on the methods from Brand-Williams et al. [43].

### Oxidative stress markers and antioxidant enzyme activity determination (BBCH 65–67; peak flowering)

To assess physiological oxidation, leaves were taken at the peak of their oxidative-stress-related metabolic activity. The plant’s oxidative status was assessed by analyzing key stress indicators and antioxidant defense enzyme activity. Hydrogen peroxide (H_2_O_2_) was determined spectrophotometrically by means of potassium iodide oxidation [44]. Lipid peroxidation was evaluated based on the amount of malondialdehyde (MDA), as measured through the TBARS assay [45]. Antioxidant enzyme activity was determined, including that of ascorbate peroxidase (APX) via measuring the decrease in absorbance at 290 nm during ascorbate autoxidation [46]; superoxide dismutase (SOD) activity was also measured based on the ability to modify the photochemical reduction of nitroblue tetrazolium (NBT) [47]. All enzyme activities were calculated per total amount of soluble proteins, which was measured by the Bradford method [48].

### Phenological and yield components determination (BBCH 65–89; from flowering to maturity)

To assess phenology and yield, various aspects of development and plant productivity were studied. The days taken from planting until 50% flowering on the main central capitulum (BBCH 65) were recorded. At BBCH-69; the end of flowering, the plant structures affecting yield potential were evaluated. In this period, two parameters were really measured where plants observe capitula per plant and the main capitulum diameter using a digital caliper. For getting the very best quality seeds, physiologically matured seeds were harvested at BBCH stages 88–89 for yield determination. This was measured in terms of the number of seeds per capitulum, total seed yield per plant, and 1000-seed weight, along with seed yield per hectare.

### HPLC analysis of silymarin and related constituents (BBCH 88–89; full maturity)

The HPLC-UV assessment of the silymarin compounds included HPLC-UV determination of the amounts of the six major silymarin components: silychristin, silydianin, silybin A/B, and isosilybin A/B, as well as total silymarin content. This was done with 10 g of dried seed powder collected in the second season when the plant achieved physiological maturity. Initially, the sample was treated with hexane for four hours to remove the fats. The samples were extracted with ethyl acetate through Soxhlet extraction for eight hours. The plant extract so obtained was then diluted and analyzed by HPLC. The analysis employed an L1 column (4.6 × 150 mm, 5 μm) under a flow rate of 1.0 mL per minute. The elution was measured at a wavelength of 288 nm. A gradient elution method was executed with mobile phase A consisting of methanol, phosphoric acid, and water in the ratio 20:0.5:80 (v/v/v). Mobile phase B was methanol with phosphoric acid and water at 80:0.5:20 (v/v/v). The gradient started at 85% A and 15% B for the first five minutes and after that shifted to 55% A and 45% B through 20 min. In this regard, the method suitability has been based on a standard of the USP, with relative retention times (RRTs) completely in the acceptable range (± 2%). The retention times of all six target flavonolignans were identical to those of the calibrated standards [49].

### Leaf and stem anatomical analysis (BBCH 61; beginning of flowering)

Samples were collected from leaves located at eighth node from the apex on the main stem during the first growing season at the beginning of the flowering stage. Immediately after collection, the samples were fixed in FAA solution (95% ethanol, 50 mL; 10 mL of formaldehyde; 5 mL of glacial acetic acid; and 35 mL of distilled water) for 48 h [50, 51]. After fixation, the samples were rinsed, dehydrated in a t-butanol series, cleared, and then embedded in paraffin wax. Transverse Sect. (20 μm) were cut on a rotary microtome, mounted on slides with Haupt’s adhesive, and stained with a crystal violet-erythrosin mixture [52]. The sections were then cleared in carbol xylene and mounted in Canada balsam. The observation was carried out using an upright light microscope (AxioPlan, Zeiss, Jena, Germany). Image processing and measurement were performed using CaseViewer 2.3 software (3DHISTECH Ltd.) [53].

### Statistical analysis

The data underwent statistical analysis using IBM SPSS Statistics version-27 (IBM Corp.-Armonk- NY- USA). Before the analysis of variance (ANOVA), we assessed the assumptions of normality and homogeneity of variance for all variables using the Shapiro-Wilk and Levene’s tests, respectively. A combined analysis of variance (ANOVA) was performed for the two growing seasons. The experimental design was a split-plot arranged in a Randomized Complete Block Design (RCBD), where biochar treatments were assigned to the main plots and α-Toco concentrations to the sub-plots. The statistical model used was: $Y_{ijkl}=\mu+S_i+R_{j(i)}+B_k+(SB)_{ik}+\varepsilon_{aijk}+T_l+(ST)_{il}+(BT)_{kl}+(SBT)_{ikl}+\varepsilon_{bijkl}$​​ where $Y_{ijkl}$ is the observed value, *µ* is the overall mean, $S_i$ is the season effect, $R_{j\left(i\right)}$is the replication nested within the season, $B_k$ is the biochar effect, ${\left(SB\right)}_{ik}$​ is the season × biochar interaction, $T_{l}$​ is the α Toco effect, ${\left(ST\right)}_{il}$​ is the season × α-Toco interaction, ${\left(BT\right)}_{kl}$​ is the interaction effect (biochar × α-Toco), ${\left(SBT\right)}_{ikl}$​ is the three-way interaction, and *εa*​ and *εb*​ are the main plot and sub-plot errors, respectively. The sources of variation included Season (df = 1), Replication (Season) (df = 4), Biochar (df = 3), Season × Biochar (df = 3), Error A (df = 12), α-Toco (df = 3), Season × α-Toco (df = 3), the Biochar × α-Toco interaction (df = 9), Season × Biochar × α-Toco (df = 9), and Error B (df = 48). We used Tukey’s Honestly Significant Difference (HSD) test to separate treatment means. A *p*-value of ≤ 0.05 was considered statistically significant. Results are presented as means ± standard error (SE). In addition, the Pearson’s correlation, heat map with hierarchical clustering and a PCA-biplot were performed.

### Results

### Growth measurements

The results indicated a clear dose-dependent positive trend in growth parameters in response to biochar and α-Toco applications (Table 4). The results showed that under salty soil conditions (ECe of 8.55 dS m⁻¹), the growth of milk thistle was affected by the rates of biochar added to the soil and leafy-spraying α-Toco levels, either alone or together. Overall, in the absence of biochar or α-Toco there was a reduction in plant height, the number of branches plant^− 1^, dry above-ground biomass plant^− 1^, root length plant^− 1^, and dry below-ground biomass plant^− 1^. Conversely, applying biochar at rates of 5 to 15 t ha^− 1^ had a positive, dose-dependent effect on all these growth responses. The highest growth improvements were seen at a rate of 10 t ha^− 1^, with increases of the former attributes by 34.3%, 96.3%, 162.9%, 117%, and 111.6%, respectively, compared to the control plants under salt stress. The treatment with 15 t ha^− 1^ also led to significant growth improvements, but the increases were slightly less than those at the 10-t rate.

**Table 4:** Effect of biochar application and α-tocopherol, and their interaction on growth measurements of milk thistle grown in (S_I_) 2023/24 and (S_II_) 2024/25 winter seasons

Treatment		Plant height	Number of branches plant^− 1^	Above-ground dry biomass plant^− 1^	Root length plant^− 1^	Below-ground dry biomass plant^− 1^
		(cm)		(g)	(cm)	(g)
Seasons (S)						
S_I_		121.1 ± 1.9b	13.1 ± 1.11b	234.6 ± 1.19b	16.5 ± 1.14b	21.3 ± 1.16b
S_II_		124.3 ± 2.2a	14.3 ± 1.16a	246.7 ± 1.19a	19.6 ± 1.14a	24.4 ± 1.15a
Biochar (Bio) t ha^− 1^						
0		105.7 ± 3.21d	8.61 ± 1.62d	130.6 ± 1.63d	10.6 ± 1.10d	13.8 ± 1.14d
5		115.1 ± 2.21c	11.7 ± 1.26c	180.3 ± 1.25c	16.6 ± 1.13c	19.4 ± 1.13c
10		142.0 ± 3.10a	16.9 ± 1.32b	343.4 ± 1.12a	23.0 ± 1.16a	29.2 ± 1.16a
15		127.8 ± 2.28b	17.5 ± 1.45a	308.3 ± 1.21b	21.9 ± 1.15b	29.0 ± 1.16b
Tocopherol (Toco) ppm						
0		107.9 ± 3.01d	11.4 ± 1.23d	179.9 ± 1.23d	13.5 ± 1.10d	19.4 ± 1.16d
50		121.8 ± 2.11c	13.1 ± 1.32c	222.4 ± 1.54c	17.2 ± 1.14c	21.6 ± 1.16c
100		127.2 ± 1.89b	14.5 ± 1.25b	245.3 ± 1.25b	19.3 ± 1.13b	23.7 ± 1.15b
150		133.7 ± 2.22a	15.8 ± 2.03a	315.0 ± 1.23a	22.1 ± 1.14a	26.5 ± 1.14a
Bio × Toco						
Bio 0	Toco 0	102.1 ± 1.41 m	7.64 ± 1.68 L	120.1 ± 1.34p	9.71 ± 1.03k	12.9 ± 1.11p
	Toco 50	104.3 ± 1.49 L	8.58 ± 1.24k	126.5 ± 1.23o	10.5 ± 1.08k	13.5 ± 1.12o
	Toco 100	106.8 ± 2.11k	8.62 ± 1.42k	132.9 ± 1.12n	10.7 ± 1.08jk	14.1 ± 1.12n
	Toco 150	109.8 ± 2.01j	9.58 ± 1.42j	142.9 ± 1.23 m	11.5 ± 1.06ij	14.6 ± 1.13 m
Bio 5	Toco 0	105.2 ± 1.97kl	10.6 ± 1.41i	164.7 ± 1.11 L	12.5 ± 1.09i	17.7 ± 1.14 L
	Toco 50	113.4 ± 2.10i	10.6 ± 1.48i	173.7 ± 1.21k	15.2 ± 1.08 h	18.8 ± 1.13k
	Toco 100	117.2 ± 1.75 h	12.1 ± 1.36 h	182.5 ± 1.32j	18.5 ± 1.06f	19.9 ± 1.13j
	Toco 150	124.4 ± 2.12 g	13.6 ± 1.25f	200.3 ± 1.21i	20.2 ± 1.05e	21.2 ± 1.12i
Bio 10	Toco 0	113.3 ± 1.75i	12.6 ± 1.47 g	209.2 ± 1.34 h	14.4 ± 1.05 h	23.4 ± 1.13 h
	Toco 50	142.5 ± 1.69c	16.6 ± 1.36d	320.1 ± 1.28d	23.8 ± 1.11c	27.3 ± 1.14e
	Toco 100	152.0 ± 1.59b	18.5 ± 1.47c	383.9 ± 1.24c	25.7 ± 1.14b	30.7 ± 1.15c
	Toco 150	160.3 ± 2.32a	19.9 ± 1.23b	460.4 ± 1.23a	28.3 ± 1.09a	35.3 ± 1.16a
Bio 15	Toco 0	111.2 ± 1.23j	14.7 ± 1.25e	225.8 ± 1.24 g	17.5 ± 1.13 g	23.8 ± 1.16 g
	Toco 50	127.0 ± 1.85f	16.6 ± 1.42d	269.1 ± 1.25f	19.4 ± 1.21ef	27.0 ± 1.15f
	Toco 100	132.8 ± 2.36e	18.6 ± 1.06c	281.9 ± 1.26e	22.3 ± 1.10d	30.2 ± 1.15d
	Toco 150	140.1 ± 1.45d	20.1 ± 1.32a	456.6 ± 1.24b	28.2 ± 1.11a	35.0 ± 1.14b

Results are presented as means ± standard error (*n* = 3). Mean values in the same column followed by a distinct lowercase letter are significantly different according to Tukey’s HSD test (*p* ≤ 0.05)

Similarly, spraying α-Toco at concentrations of 50, 100, and 150 ppm significantly improved all the previously mentioned growth parameters of milk thistle, compared to the non-sprayed plants (control group, which received only distilled water). The results indicated that the highest concentration of α-Toco, at 150 ppm, produced the best improvements. The treatment led to increases of 23.9%, 38.6%, 75.1%, 63.7%, and 36.6% in plant height, number of branches plant^− 1^, above-ground biomass, root length, and below-ground biomass, respectively, compared to other α-Toco concentrations and the control plants (Table 4).

The analysis showed that the interaction between biochar and α-Toco had a significant effect on the growth of milk thistle grown in salty soil (Table 4). The combined application of 10 t ha^− 1^ biochar and 150 ppm α-Toco resulted in the best performance. Compared to the control group, which had no biochar or α-Toco, this combination resulted in increases of about 57%, 160.5%, 283.3%, 191.5%, and 173.6% for plant height, number of branches plant^− 1^, above-ground biomass, root length, and below-ground biomass, respectively.

### Photosynthetic pigment content and physiological measurements

Data in Table 5 showed that under saline soil conditions, the levels of milk thistle’s photosynthetic pigments, including Chl *a*, Chl *b*, and Car, as well as key physiological indicators like RWC and MSI, were at their lowest. At the same time, the ratio of (Chl *a* + *b*)/Car was the highest. On the other hand, adding biochar to the soil had a strong positive effect on the photosynthetic pigments and the water status of the milk thistle. At a rate of 10 t ha⁻¹, the levels of Chl *a*, Chl *b*, Car, RWC, and MSI increased by 10.9%, 19.3%, 27.5%, 12.0% and 11.9%, respectively. However, the (Chl *a* + *b*)/Car ratio dropped by 8.9% compared to un-amended (control) soil. The 15-t ha⁻¹ treatment maintained significant effects, though slightly less pronounced than the 10-t ha⁻¹ treatment over the two growing seasons.

**Table 5:** Effect of biochar application and α-tocopherol, and their interaction on photosynthetic pigment content and physiological measurements of milk thistle grown in (S_I_) 2023/24 and (S_II_) 2024/25 winter seasons

Treatment		Photosynthetic pigment content				Physiological measurements	
		Chl a	Chl b	Car	Total chl /Car ratio	RWC	MSI
		(mg g-1FW)				(%)	
Seasons (S)							
S_I_		0.96 ± 0.002b	0.63 ± 0.003a	0.46 ± 0.002a	3.49 ± 0.02a	79.7 ± 0.13b	76.3 ± 0.12b
S_II_		0.99 ± 0.002a	0.64 ± 0.003a	0.47 ± 0.002a	3.51 ± 0.02a	81.9 ± 0.13a	78.2 ± 0.12a
Biochar (Bio) t ha^− 1^							
0		0.92 ± 0.003d	0.57 ± 0.004c	0.40 ± 0.003c	3.72 ± 0.03c	75.7 ± 0.14d	72.6 ± 0.11d
5		0.96 ± 0.003c	0.64 ± 0.004b	0.46 ± 0.003b	3.54 ± 0.03b	79.3 ± 0.15c	75.7 ± 0.14c
10		1.02 ± 0.003a	0.68 ± 0.004a	0.51 ± 0.003a	3.39 ± 0.03a	84.8 ± 0.15a	81.2 ± 0.11a
15		1.00 ± 0.003b	0.66 ± 0.004a	0.50 ± 0.003a	3.37 ± 0.03a	83.4 ± 0.14b	79.6 ± 0.12b
Tocopherol (Toco) ppm							
0		0.91 ± 0.003d	0.56 ± 0.004d	0.39 ± 0.003d	3.81 ± 0.03c	77.1 ± 0.16d	73.7 ± 0.13d
50		0.95 ± 0.003c	0.61 ± 0.004c	0.45 ± 0.003c	3.48 ± 0.03b	79.8 ± 0.17c	77.0 ± 0.14c
100		1.00 ± 0.003b	0.67 ± 0.004b	0.49 ± 0.003b	3.40 ± 0.03ab	81.9 ± 0.15b	78.6 ± 0.12b
150		1.05 ± 0.003a	0.71 ± 0.004a	0.53 ± 0.003a	3.33 ± 0.03a	84.4 ± 0.14a	79.8 ± 0.12a
Bio × Toco							
Bio 0	Toco 0	0.87 ± 0.01i	0.52 ± 0.01i	0.35 ± 0.01k	3.98 ± 0.05 g	72.4 ± 0.17n	69.7 ± 0.15o
	Toco 50	0.91 ± 0.01gh	0.54 ± 0.01hi	0.38 ± 0.01jk	3.83 ± 0.05 g	74.7 ± 0.16 m	71.6 ± 0.14n
	Toco 100	0.93 ± 0.01 fg	0.58 ± 0.01f-h	0.43 ± 0.01hi	3.52 ± 0.05c-f	76.7 ± 0.17k	73.5 ± 0.14 L
	Toco 150	0.96 ± 0.01de	0.63 ± 0.01de	0.45 ± 0.01f-h	3.54 ± 0.05d-f	78.8 ± 0.17i	75.7 ± 0.14i
Bio 5	Toco 0	0.90 ± 0.01 h	0.55 ± 0.01 g-i	0.39 ± 0.01j	3.77 ± 0.05 fg	75.8 ± 0.16 L	72.5 ± 0.14 m
	Toco 50	0.94 ± 0.01ef	0.59 ± 0.01ef	0.44 ± 0.01gh	3.50 ± 0.05c-f	78.1 ± 0.19j	74.7 ± 0.16k
	Toco 100	0.97 ± 0.01d	0.67 ± 0.01 cd	0.48 ± 0.01ef	3.42 ± 0.05d-f	80.1 ± 0.18 h	76.8 ± 0.15 h
	Toco 150	1.04 ± 0.01c	0.73 ± 0.01ab	0.51 ± 0.01c-e	3.45 ± 0.05c-f	83.1 ± 0.14e	78.7 ± 0.15f
Bio 10	Toco 0	0.94 ± 0.01ef	0.59 ± 0.01 fg	0.41 ± 0.01ij	3.78 ± 0.05 fg	79.0 ± 0.15i	75.2 ± 0.14j
	Toco 50	0.97 ± 0.01de	0.64 ± 0.01d	0.47 ± 0.01 fg	3.39 ± 0.05d-f	84.4 ± 0.17c	82.3 ± 0.16c
	Toco 100	1.07 ± 0.01b	0.74 ± 0.01ab	0.55 ± 0.01bc	3.31 ± 0.05d-g	87.0 ± 0.17b	83.3 ± 0.13b
	Toco 150	1.12 ± 0.01a	0.75 ± 0.01a	0.61 ± 0.01a	3.09 ± 0.05a	88.9 ± 0.18a	84.1 ± 0.13a
Bio 15	Toco 0	0.94 ± 0.01ef	0.56 ± 0.01f-i	0.40 ± 0.01ij	3.73 ± 0.05e-g	81.1 ± 0.19 g	77.3 ± 0.14 g
	Toco 50	0.96 ± 0.01de	0.65 ± 0.01d	0.51 ± 0.01de	3.18 ± 0.05ab	81.8 ± 0.16f	79.5 ± 0.15e
	Toco 100	1.04 ± 0.01c	0.70 ± 0.01bc	0.52 ± 0.01 cd	3.34 ± 0.05d-g	83.9 ± 0.14d	80.7 ± 0.14d
	Toco 150	1.07 ± 0.01b	0.74 ± 0.01a	0.56 ± 0.01b	3.24 ± 0.05a-c	87.0 ± 0.17b	80.8 ± 0.13d

Results are presented as means ± standard error (*n* = 3). Mean values in the same column followed by a distinct lowercase letter are significantly different according to Tukey’s HSD test (*p* ≤ 0.05)

*Chl a *Chlorophyll *a*, *Chl b *chlorophyll *b* , *Car* total carotenoid , *Total chl /Car ratio* total chlorophyll/ total carotenoid, *RWC *relative water content, and *MSI* membrane stability index

Moreover, under saline soil conditions, leafy-spraying with α-Toco in a dose-dependent manner enhanced the above-mentioned pigment and physiological parameters significantly compared with the control group (Table 5). When α-Toco was supplemented from 0 (non-treated control) up to 150 ppm (highest level), Chl *a* increased by 15.4%, Chl *b* by 26.8%, and Car by 35.9%. RWC went up by 9.5%, and MSI increased by 8.3%. The identical 150-treatment caused the biggest drop in the (Chl *a* + *b*)/Car ratio, which decreased by 12.6%.

Co-application of biochar and α-Toco demonstrated additive interactions. The application of 10 t ha^− 1^ biochar combined with 150 ppm α-Toco gave the best results for all the measured factors over the two growing seasons, as shown in Table 5. Notably, this combination achieved the highest observed increases for Chl *a* (28.7%), Chl *b* (44.2%), Car (74.3%), RWC (22.8%), and MSI (20.7) when compared to biochar 0 control at the same α-Toco concentration. Furthermore, the (Chl *a* + *b*)/Car ratio exhibited a decline of 22.4%.

### Nutrient contents

The results indicated that milk thistle plants under salt stress, when treated with biochar at 10 or 15 t ha⁻¹ (Table 6), responded positively. Biochar significantly improved leaf nutrient uptake and accumulation in a dose-dependent manner. Specifically, increasing biochar application from 0 to 10 t ha⁻¹ resulted in a 22.2% increase in N, 49.8% in P, 18.6% in K, 14.2% in Mg, and 14.1% in Zn. Conversely, Na concentration decreased by 24.7%. The 15-t ha⁻¹ treatment further increased Mg and Zn levels by 19.3% and 15.9%, respectively, while maintaining Na at the lowest levels (28.2%) compared to the un-amended control (0 t ha⁻¹).

**Table 6:** Effect of biochar application and α-tocopherol, and their interaction on leaf nutrient contents of milk thistle grown in (S_I_) 2023/24 and (S_II_) 2024/25 winter seasons

Treatment		*N*	*P*	K	Mg	Zn	Na
		(mg g^− 1^ DW)					
Seasons (S)							
S_I_		34.4 ± 0.32b	6.91 ± 0.13b	46.8 ± 0.52b	4.67 ± 0.06b	4.43 ± 0.04b	6.44 ± 0.04b
S_II_		34.9 ± 0.24a	6.94 ± 0.13a	47.8 ± 0.42a	4.70 ± 0.06a	4.48 ± 0.04a	6.25 ± 0.03a
Biochar (Bio) t ha^− 1^							
0		30.6 ± 0.29d	5.44 ± 0.11d	42.4 ± 0.83d	4.30 ± 0.05d	4.10 ± 0.03d	7.69 ± 0.02d
5		33.5 ± 0.56c	6.43 ± 0.12c	46.7 ± 0.85c	4.40 ± 0.04c	4.30 ± 0.04c	6.38 ± 0.03c
10		37.4 ± 0.58a	8.15 ± 0.14a	50.3 ± 0.83a	4.91 ± 0.05b	4.68 ± 0.03b	5.79 ± 0.03b
15		37.1 ± 0.48b	7.68 ± 0.12b	49.9 ± 0.81b	5.13 ± 0.05a	4.75 ± 0.02a	5.52 ± 0.02a
Tocopherol (Toco) ppm							
0		33.2 ± 0.43d	6.54 ± 0.11d	46.0 ± 0.73d	4.30 ± 0.06d	4.27 ± 0.03d	6.71 ± 0.04d
50		34.3 ± 0.45c	6.66 ± 0.12c	46.7 ± 0.71c	4.53 ± 0.07c	4.36 ± 0.02c	6.47 ± 0.05c
100		35.0 ± 0.39b	7.09 ± 0.10b	47.8 ± 0.74b	4.68 ± 0.06b	4.47 ± 0.02b	6.25 ± 0.05b
150		36.2 ± 0.42a	7.41 ± 0.11a	48.9 ± 0.83a	5.23 ± 0.04a	4.73 ± 0.01a	5.95 ± 0.05a
Bio × Toco							
Bio 0	Toco 0	29.6 ± 0.56 m	5.03 ± 0.07 L	40.8 ± 0.36 m	4.13 ± 0.03o	4.03 ± 0.02o	8.16 ± 0.06p
	Toco 50	30.3 ± 0.49 L	5.15 ± 0.06k	42.0 ± 0.21 L	4.26 ± 0.03 L	4.07 ± 0.03n	7.92 ± 0.04o
	Toco 100	31.2 ± 0.44k	5.58 ± 0.05j	42.8 ± 0.39k	4.38 ± 0.03j	4.11 ± 0.04 m	7.67 ± 0.07n
	Toco 150	31.5 ± 0.43k	6.01 ± 0.06i	43.9 ± 0.62j	4.43 ± 0.03i	4.17 ± 0.02 L	7.02 ± 0.07 m
Bio 5	Toco 0	32.7 ± 0.39j	6.04 ± 0.05i	45.6 ± 0.42i	4.20 ± 0.02n	4.21 ± 0.01k	6.72 ± 0.05 L
	Toco 50	33.3 ± 0.38i	6.16 ± 0.07 h	46.0 ± 0.52 h	4.33 ± 0.05k	4.29 ± 0.02j	6.44 ± 0.06k
	Toco 100	33.8 ± 0.42 h	6.63 ± 0.06 g	47.2 ± 0.50 g	4.51 ± 0.04 h	4.33 ± 0.03i	6.24 ± 0.05j
	Toco 150	34.3 ± 0.43 g	6.91 ± 0.05f	48.2 ± 0.52f	4.58 ± 0.03f	4.37 ± 0.02 h	6.14 ± 0.05i
Bio 10	Toco 0	35.5 ± 0.52e	8.05 ± 0.06b	48.8 ± 0.39e	4.23 ± 0.04 m	4.41 ± 0.04 g	6.02 ± 0.03 h
	Toco 50	37.1 ± 0.42c	7.67 ± 0.05d	49.5 ± 0.42d	4.53 ± 0.09 g	4.45 ± 0.03e	5.93 ± 0.14f
	Toco 100	37.5 ± 0.53b	8.08 ± 0.06b	50.9 ± 0.43b	4.93 ± 0.07d	4.71 ± 0.04c	5.79 ± 0.12e
	Toco 150	39.5 ± 0.38a	8.80 ± 0.06a	51.8 ± 0.36a	5.94 ± 0.06b	5.13 ± 0.03b	5.44 ± 0.10c
Bio 15	Toco 0	34.9 ± 0.36f	7.06 ± 0.05e	48.6 ± 0.46e	4.64 ± 0.08e	4.43 ± 0.05f	5.94 ± 0.11 g
	Toco 50	36.4 ± 0.51d	7.67 ± 0.07d	49.3 ± 0.41d	5.00 ± 0.07c	4.63 ± 0.06d	5.62 ± 0.08d
	Toco 100	37.5 ± 0.49b	8.08 ± 0.06b	50.1 ± 0.36c	4.93 ± 0.05d	4.71 ± 0.04c	5.32 ± 0.07b
	Toco 150	39.5 ± 0.39a	7.93 ± 0.05c	51.7 ± 0.34a	5.98 ± 0.03a	5.23 ± 0.04a	5.22 ± 0.04a

Results are presented as means ± standard error (*n* = 3). Mean values in the same column followed by a distinct lowercase letter are significantly different according to Tukey’s HSD test (*p* ≤ 0.05)

*N* Nitrogen, *P* phosphorus, *K* potassium, *Mg* magnesium, *Zn* zinc, and *Na* sodium

Over the two growing seasons, foliar spraying with α-Toco improved the nutrient balance in milk thistle leaves grown in salty soil (Table 6). In particular, increasing α-Toco levels consistently raised the levels of leaf nutrients (N, P, K, Mg, and Zn) while also lowering the levels of Na, compared to the untreated control. Leafy-feeding with 100 or 150 ppm α-Toco resulted in statistically significant increases of 5.4% or 9.0% for N, 8.4% or 13.3% for P, 3.9% or 6.3% for K, 8.8% or 21.6% for Mg, and 4.7% or 10.8% for Zn, alongside a reduction in Na + of 6.9% or 11.3%, respectively, compared to the untreated control.

As for the biochar × α-Toco interaction, the results presented in Table 6 indicate that this mixture produced the highest response on leaf nutrient contents. The maximum N, P and K contents were obtained with the application of 10-t ha^‒1^ biochar along with 150 ppm of α-Toco. This combination yielded substantial increases over the absolute control (biochar 0 t ha^− 1^ and α-Toco 0 ppm) of 33.5% for N, a remarkable 75% for P, and 27% for K. Similarly, Mg and Zn levels were also highest when using 15 t ha^− 1^ of biochar and 150 ppm of α-Toco, which boosted Mg by 44.8% and Zn by 29.8% compared to the lowest interaction. Additionally, this mixture was the most effective at reducing Na buildup, lowering Na content by 36% compared to the control treatment.

### Leaf phytochemical contents

Data on all the phytochemicals analyzed in Table 7 showed that the biochar treatment had a significant (*p* < 0.05) positive effect in a dose-dependent manner. At the rate of 10 t ha⁻¹, a significant increase was observed as compared to the control (0 t ha⁻¹). TSS increased by 73.0%, while TPC and TFC were up by 49.5% and 49.9%, respectively. The free proline content was also decreased by 27.2%. At the highest dose of 15 t ha⁻¹, this positive trend was further enhanced, resulting in outstanding TSS (89.1%), TPC (60.4%), and TFC (61.1%) levels, along with a significant 37.6% reduction in free proline. In the meantime, the antioxidant activity was greatly improved with a reduction of IC_50_ value by 38.7% at 10 t ha⁻¹ and 45.6% at 15 t ha^− 1^.

**Table 7:** Effect of biochar application and α-tocopherol, and their interaction on leaf phytochemical contents of milk thistle grown in (S_I_) 2023/24 and (S_II_) 2024/25 winter seasons

Treatment		TSS	TPC	TFC	Free proline	EAT	IC_50_
		(mg g^− 1^ DW)				(µg g^− 1^ DW)	(µg mL^− 1^)
Seasons (S)							
S_I_		19.3 ± 0.21b	25.5 ± 0.32b	12.4 ± 0.13b	1.10 ± 0.08b	151.1 ± 0.03b	79.8 ± 0.01b
S_II_		21.6 ± 0.22a	28.5 ± 0.32a	13.8 ± 0.14a	0.96 ± 0.07a	158.8 ± 0.03a	73.4 ± 0.01a
Biochar (Bio) t ha^− 1^							
0		13.7 ± 0.32d	20.2 ± 0.32d	9.81 ± 0.15d	1.25 ± 0.09d	130.7 ± 0.02d	99.9 ± 0.01d
5		18.6 ± 0.33c	25.1 ± 0.31c	12.2 ± 0.16c	1.18 ± 0.08c	151.6 ± 0.02c	90.9 ± 0.01c
10		23.7 ± 0.31b	30.2 ± 0.25b	14.7 ± 0.15b	0.91 ± 0.09b	166.5 ± 0.03b	61.2 ± 0.01b
15		25.9 ± 0.29a	32.4 ± 0.30a	15.8 ± 0.14a	0.78 ± 0.07a	171.0 ± 0.02a	54.4 ± 0.01a
Tocopherol (Toco) ppm							
0		17.4 ± 0.28d	23.9 ± 0.26d	11.7 ± 0.15d	1.15 ± 0.06d	129.8 ± 0.01d	98.7 ± 0.01d
50		19.3 ± 0.26c	25.8 ± 0.23c	12.5 ± 0.14c	1.07 ± 0.08c	151.4 ± 0.02c	79.5 ± 0.01c
100		21.3 ± 0.31b	27.8 ± 0.20b	13.5 ± 0.15b	1.01 ± 0.07b	162.9 ± 0.03b	68.3 ± 0.01b
150		23.9 ± 0.23a	30.4 ± 0.21a	14.8 ± 0.13a	0.89 ± 0.07a	175.7 ± 0.04a	59.8 ± 0.01a
Bio × Toco							
Bio 0	Toco 0	12.2 ± 0.11n	18.7 ± 0.19n	9.10 ± 0.16n	1.30 ± 0.07n	111.8 ± 0.02 m	110.8 ± 0.00n
	Toco 50	13.1 ± 0.12 m	19.6 ± 0.20 m	9.52 ± 0.17 m	1.27 ± 0.06 m	127.9 ± 0.03 L	105.7 ± 0.00 m
	Toco 100	14.2 ± 0.11 L	20.7 ± 0.21 L	10.1 ± 0.16 L	1.24 ± 0.02 L	137.0 ± 0.02j	95.0 ± 0.01 L
	Toco 150	15.2 ± 0.10k	21.7 ± 0.22k	10.5 ± 0.14k	1.21 ± 0.08k	146.1 ± 0.03i	88.0 ± 0.01j
Bio 5	Toco 0	15.9 ± 0.09j	22.5 ± 0.31j	10.9 ± 0.16j	1.22 ± 0.06kl	132.5 ± 0.02k	107.5 ± 0.00 m
	Toco 50	17.1 ± 0.09i	23.6 ± 0.25i	11.5 ± 0.16i	1.19 ± 0.08jk	148.9 ± 0.04i	93.1 ± 0.01 L
	Toco 100	19.7 ± 0.13 h	26.2 ± 0.21 h	12.7 ± 0.17 h	1.16 ± 0.06j	155.0 ± 0.05 h	83.4 ± 0.00 h
	Toco 150	21.7 ± 0.22 g	28.2 ± 0.23 g	13.7 ± 0.09 g	1.13 ± 0.06i	170.1 ± 0.04e	79.5 ± 0.01 g
Bio 10	Toco 0	19.7 ± 0.21 h	26.2 ± 0.24 h	12.7 ± 0.09 h	1.06 ± 0.07 h	135.9 ± 0.02j	90.7 ± 0.01k
	Toco 50	22.0 ± 0.14f	28.5 ± 0.24f	13.9 ± 0.08f	0.96 ± 0.08f	162.1 ± 0.03 g	61.6 ± 0.01f
	Toco 100	24.3 ± 0.15e	30.8 ± 0.23e	15.0 ± 0.09e	0.91 ± 0.06e	177.2 ± 0.02d	52.6 ± 0.01d
	Toco 150	28.7 ± 0.14b	35.2 ± 0.25b	17.1 ± 0.10b	0.69 ± 0.05b	190.8 ± 0.01b	39.6 ± 0.01b
Bio 15	Toco 0	21.9 ± 0.12f	28.5 ± 0.18f	13.8 ± 0.11f	1.00 ± 0.04 g	139.0 ± 0.02j	85.7 ± 0.01i
	Toco 50	24.9 ± 0.21d	31.4 ± 0.11d	15.3 ± 0.12d	0.88 ± 0.07d	166.8 ± 0.02f	57.5 ± 0.01e
	Toco 100	27.1 ± 0.13c	33.6 ± 0.12c	16.3 ± 0.13c	0.73 ± 0.03c	182.2 ± 0.01c	42.2 ± 0.01c
	Toco 150	29.9 ± 0.15a	36.4 ± 0.21a	17.7 ± 0.11a	0.51 ± 0.05a	195.8 ± 0.03a	32.2 ± 0.01a

Results are presented as means ± standard error (*n* = 3). Mean values in the same column followed by a distinct lowercase letter are significantly different according to Tukey’s HSD test (*p* ≤ 0.05)

*TSS* Total soluble sugars, *TPC* total phenolic content, *TFC* total flavonoid content, *EAT* endogenous alpha-tocopherol, and *IC*_*50*_ half-maximal inhibitory levels

Likewise, the leafy spraying with α-Toco resulted in a significant increase in the phytochemical components in a dose-dependent way. At 100 ppm, significant increments with respect to the non-sprayed plants (control 0 ppm) were observed: TSS increased by 22.4%, TPC by 16.3%, and TFC by 15.4%. At the highest dose of 150 ppm, a superior effect was noted, with a 37.4% increase in TSS and increases of 27.2% and 26.5% in TPC and TFC, respectively. At the same time, the level of free proline reduced by 12.2% at 100 ppm and by 22.6% at 150 ppm. Concurrently, the antioxidant activity was also enhanced, with the IC_50_ value decreasing by 30.8% at 100 ppm and by 39.4% at 150 ppm.

The biochar × α-Toco interaction was also significant during both growing seasons, as shown in Table 7. The best results were obtained for 15 t ha⁻¹ biochar + 150 ppm α-Toco. With the best treatment dose, there were substantial improvements in all these attributes as compared with the untreated control: TSS, TPC, TFC, and EAT increased by 145.1, 94.7, 94.5, and 75.1%, respectively. Along with this, the content of free proline was reduced by 60.8%, and the IC_50_ value was reduced by 70.9%. The second-best performance came from combining 15 t ha⁻¹ biochar with 100 ppm α-Toco. This treatment also resulted in significant increases of 135.2% for TSS, 88.2% for TPC, 87.9% for TFC, and 70.7% for EAT. It was also responsible for a 46.9% decrease in free proline and a 64.3% decrease in IC_50_.

### Oxidative stress markers and antioxidant enzyme activity

The addition of biochar to the soil had a strong positive effect on oxidative stress with a clear dose-response trend (Table 8). The application of 10 t ha⁻¹ biochar brought about significant biochemical alterations in comparison with the control (0 t ha⁻¹). Notably, H₂O₂ and MDA levels were significantly reduced by 32.5% and 41.1%, respectively. This was accompanied by 21.9% and 24.7% higher APX and SOD activity, respectively. This favorable response was further enhanced at the maximum application rate, 15 t ha⁻¹, which induced further reduction in H₂O₂ and MDA (38.5% and 43.8%) and further stimulation in APX and SOD activities (23.2% and 29.5%).

**Table 8:** Effect of biochar application and α-tocopherol, and their interaction on oxidative stress markers and antioxidant enzyme activity of milk thistle grown in (S_I_) 2023/24 and (S_II_) 2024/25 winter seasons

Treatment		Oxidative stress markers		Antioxidant enzyme activity	
		H_2_O_2_	MDA	APX	SOD
		(µmol g-1FW)		(µmol mg-1Protein)	
Seasons (S)					
S_I_		1.96 ± 0.12b	0.58 ± 0.13b	6.39 ± 0.01b	4.19 ± 0.01b
S_II_		1.73 ± 0.11a	0.52 ± 0.13a	7.48 ± 0.01a	5.24 ± 0.01a
Biochar (Bio) t ha^− 1^					
0		2.34 ± 0.10d	0.73 ± 0.13d	6.08 ± 0.01d	4.13 ± 0.01d
5		2.02 ± 0.09c	0.63 ± 0.11c	6.75 ± 0.01c	4.23 ± 0.01c
10		1.58 ± 0.12b	0.43 ± 0.12b	7.41 ± 0.01b	5.15 ± 0.01b
15		1.44 ± 0.14a	0.41 ± 0.13a	7.49 ± 0.01a	5.35 ± 0.01a
Tocopherol (Toco) ppm					
0		2.12 ± 0.16d	0.63 ± 0.13d	6.52 ± 0.01d	4.19 ± 0.01d
50		1.91 ± 0.15c	0.57 ± 0.12c	6.79 ± 0.01c	4.53 ± 0.01c
100		1.76 ± 0.11b	0.52 ± 0.12b	7.03 ± 0.01b	4.88 ± 0.01b
150		1.59 ± 0.11a	0.47 ± 0.14a	7.39 ± 0.01a	5.26 ± 0.01a
Bio × Toco					
Bio 0	Toco 0	2.61 ± 0.11n	0.82 ± 0.12 L	5.76 ± 0.01k	3.75 ± 0.01 L
	Toco 50	2.41 ± 0.12 m	0.76 ± 0.06k	5.94 ± 0.01j	4.08 ± 0.01j
	Toco 100	2.22 ± 0.05k	0.69 ± 0.05j	6.24 ± 0.01i	4.25 ± 0.00i
	Toco 150	2.11 ± 0.11j	0.65 ± 0.11i	6.39 ± 0.00hi	4.45 ± 0.00gh
Bio 5	Toco 0	2.31 ± 0.09 L	0.74 ± 0.12k	6.50 ± 0.01gh	3.87 ± 0.01k
	Toco 50	2.01 ± 0.11i	0.62 ± 0.11hi	6.62 ± 0.01 g	4.16 ± 0.00ij
	Toco 100	1.92 ± 0.04 h	0.59 ± 0.12 h	6.80 ± 0.01f	4.39 ± 0.00 h
	Toco 150	1.85 ± 0.05 g	0.55 ± 0.12 g	7.09 ± 0.01e	4.51 ± 0.00 fg
Bio 10	Toco 0	1.81 ± 0.12 fg	0.50 ± 0.10f	6.88 ± 0.01f	4.56 ± 0.01f
	Toco 50	1.72 ± 0.11e	0.45 ± 0.11de	7.28 ± 0.01d	4.67 ± 0.01e
	Toco 100	1.62 ± 0.12d	0.43 ± 0.12 cd	7.51 ± 0.01c	5.32 ± 0.01c
	Toco 150	1.18 ± 0.13a	0.36 ± 0.14ab	7.97 ± 0.01b	6.06 ± 0.01a
Bio 15	Toco 0	1.76 ± 0.12ef	0.47 ± 0.12d-f	6.95 ± 0.01ef	4.61 ± 0.01ef
	Toco 50	1.50 ± 0.15c	0.47 ± 0.11ef	7.33 ± 0.01d	5.19 ± 0.01d
	Toco 100	1.29 ± 0.11b	0.39 ± 0.04bc	7.57 ± 0.01c	5.56 ± 0.01b
	Toco 150	1.21 ± 0.12a	0.33 ± 0.05a	8.13 ± 0.01a	6.04 ± 0.01a

Results are presented as means ± standard error (*n* = 3). Mean values in the same column followed by a distinct lowercase letter are significantly different according to Tukey’s HSD test (*p* ≤ 0.05)

*H*_*2*_*O*_*2*_ Hydrogen peroxide, *MDA *malondialdehyde, *APX *ascorbate peroxidase, and *SOD *superoxide dismutase

Likewise, the foliar application of α-Toco significantly mitigated oxidative stress markers in milk thistle plants grown in salty soil throughout the two growing seasons (Table 8). The accumulation of H₂O₂ and MDA was inhibited by 17.0% and 17.5% at the dose of 100 ppm, respectively. Simultaneously, there was an induction of a 7.8% and 16.5% increase in APX and SOD activities, respectively. At the highest level (150 ppm), there was a further reduction in H₂O₂ by 25.0% and MDA by 25.4% as well as an enhancement in APX and SOD by 13.3% and 25.5%, respectively.

The combination of biochar and α-Toco had a synergistic effect on attenuating milk thistle’s oxidative stress. The most effective treatment, 15 t ha⁻¹ biochar combined with 150 ppm α-Toco, decreased H₂O₂ and MDA contents by 53.6% and 59.8%, respectively, and enhanced APX and SOD enzyme activities by 41.2% and 61.1%, compared with the untreated control. The runner-up, 10 t ha⁻¹ biochar with 150 ppm α-Toco, similarly reduced H₂O₂ and MDA by 54.8% and 56.1% and enhanced APX and SOD activities by 38.4 and 61.6%.

### Phenological and yield components

**Fig. 2:**
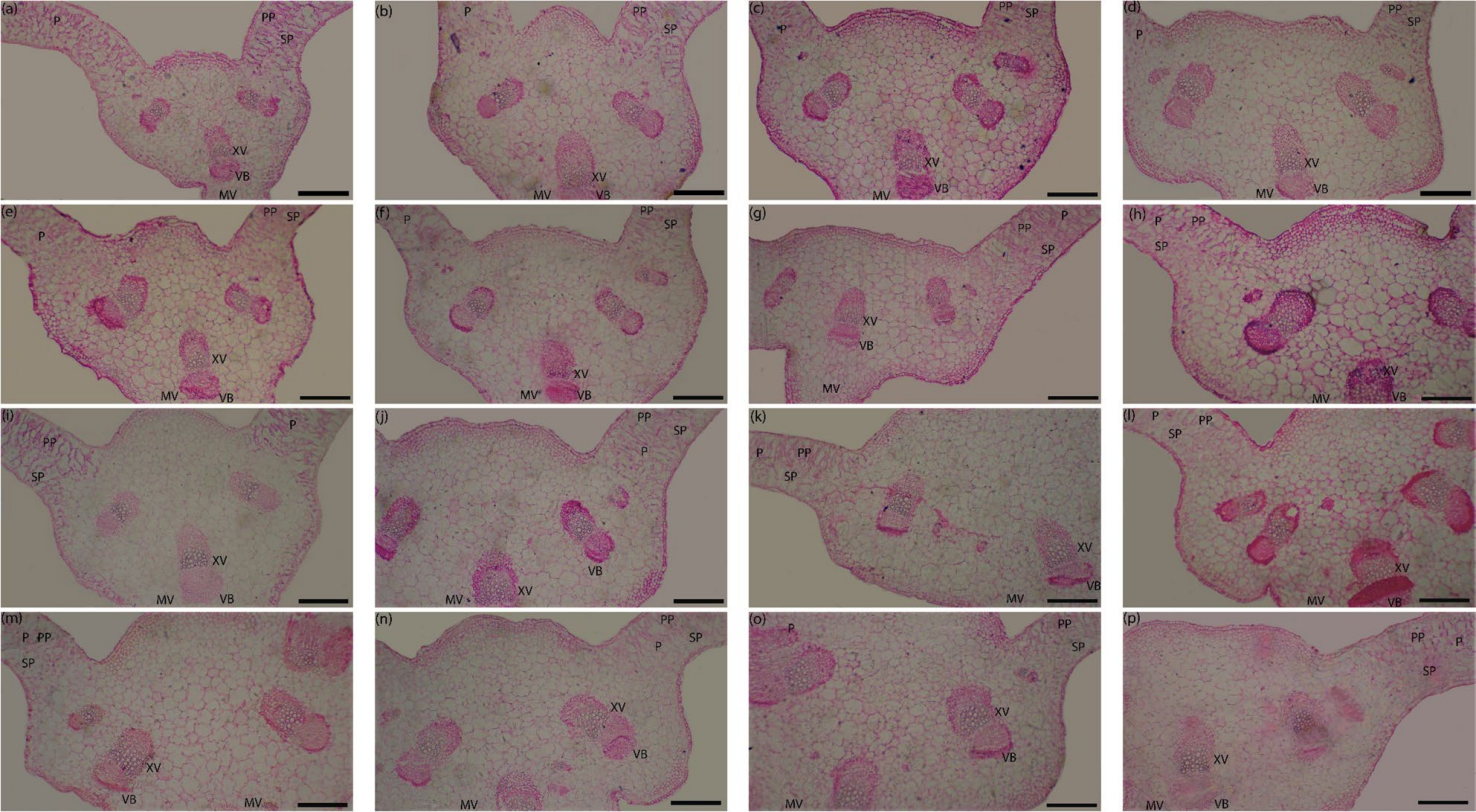
Effect of biochar application and α-tocopherol, and their interaction on transections in the midvein of leaves of milk thistle grown in (SI) 2023/24 winter season. Sponge parenchyma (SP), plastid parenchyma (PP), xylem vessels (XV), vascular bundle (VB) of the midvein (MV). Bars = 500 μm. Bio 0 × Toco 0 (**a**), Bio 0 × Toco 50 (**b**), Bio 0 × Toco 100 (**c**), Bio 0 × Toco 150 (**d**), Bio 5 × Toco 0 (**e**), Bio 5 × Toco 50 (**f**), Bio 5 × Toco 100 (**g**), Bio 5 × Toco 150 (**h**), Bio 10 × Toco 0 (**i**), Bio 10 × Toco 50 (**j**), Bio 10 × Toco 100 (**k**), Bio 10 × Toco 150 (**l**), Bio 15 × Toco 0 (**m**), Bio 15 × Toco 50 (**n**), Bio 15 × Toco 100 (**o**), Bio 15 × Toco 150 (**p**)

The results showed that biochar addition to the saline soil substrate progressively promoted, in a dose-dependent way, the phenological and yield traits (Table 9). Noticeable positive effects were already evident at a dose of 10 t ha⁻¹, leading to earlier flowering by 8.1% compared to the control (0 t ha⁻¹). Simultaneously, all yield components were significantly increased, including number of capitula plant^− 1^ by 96.0%, diameter of the main capitulum by 58.4%, number of seeds capitulum^− 1^ by 18.2%, seed yield plant^− 1^ by 79.8%, and 1000-seed weight by 11.9%. As in Fig. 2, relative to the untreated control that yielded 0.93 t ha^− 1^, biochar application of 10-t ha^− 1^ enhanced the seed yield to 1.68 t ha^− 1^, representing a significant improvement of 80.65%. This favorable trend was even stronger at the highest biochar application rate of 15 t ha⁻¹. This superior treatment accelerated flowering by 9.4% and generated a dramatic change in the yield structure, with the number of capitula plant^− 1^ more than doubling (103.5%), the main capitulum diameter increasing 60.3%, and the number of seeds capitulum^− 1^ rising 21.4%. This culminated in an exceptional increment of 81.0% in seed yield plant^− 1^, while a 12.1% enhancement in 1000-seed weight was a constant feature. A further increase in the rate of application to 15-t ha^− 1^ resulted in a small but statistically significant seed yield of 1.69 t ha^− 1^ which is an 81.72% improvement over the control.

**Table 9:** Effect of biochar application and α-tocopherol, and their interaction on phenological and yield components of milk thistle grown in (S_I_) 2023/24 and (S_II_) 2024/25 winter seasons

Treatment		Days 50% flowering	Number of capitula plant^− 1^	Main capitulum diameter	Number of seeds capitulum^− 1^	seed yield plant^− 1^	1000-seed weight
		(day)		(cm)		(g)	
Seasons (S)							
S_I_		112.4 ± 2.11b	26.3 ± 1.25b	4.60 ± 0.42b	149.1 ± 0.22b	47.4 ± 1.02b	20.6 ± 0.22b
S_II_		109.4 ± 2.31a	28.7 ± 1.26a	5.71 ± 0.42a	154.8 ± 0.23a	50.5 ± 1.11a	21.7 ± 0.23a
Biochar (Bio) t ha^− 1^							
0		117.5 ± 2.23d	17.3 ± 1.42d	3.73 ± 0.55d	136.7 ± 0.31d	33.6 ± 1.15d	19.8 ± 0.24d
5		111.9 ± 2.22c	23.6 ± 1.51c	5.02 ± 0.56c	143.8 ± 0.32c	40.9 ± 2.51c	20.4 ± 0.23c
10		107.9 ± 3.21b	33.9 ± 2.01b	5.91 ± 0.55b	161.6 ± 0.32b	60.4 ± 2.12b	22.16 ± 0.22b
15		106.5 ± 2.23a	35.2 ± 1.62a	5.98 ± 0.45a	165.9 ± 0.32a	60.8 ± 1.56a	22.20 ± 0.24a
Tocopherol (Toco) ppm							
0		114.8 ± 2.23d	22.9 ± 1.51d	4.74 ± 0.67d	145.2 ± 0.26d	43.3 ± 2.11d	20.8 ± 0.25d
50		112.4 ± 1.59c	26.3 ± 1.43c	4.94 ± 0.39c	149.7 ± 0.27c	47.0 ± 2.32c	21.0 ± 0.49c
100		110.7 ± 1.98b	29.1 ± 1.32b	5.22 ± 0.51b	153.5 ± 0.25b	50.0 ± 2.41b	21.3 ± 0.38b
150		105.9 ± 3.01a	31.7 ± 1.42a	5.73 ± 0.42a	159.5 ± 0.28a	55.4 ± 2.10a	21.6 ± 0.38a
Bio × Toco							
Bio 0	Toco 0	118.8 ± 1.95n	15.4 ± 1.33 L	3.19 ± 0.43 m	135.8 ± 0.32o	32.1 ± 1.87o	19.6 ± 0.35j
	Toco 50	118.3 ± 1.89n	17.2 ± 1.21k	3.50 ± 0.44 L	136.4 ± 0.21no	33.1 ± 1.92n	19.8 ± 0.41i
	Toco 100	116.8 ± 2.11 m	17.3 ± 1.22k	3.80 ± 0.36k	136.7 ± 0.20mn	34.1 ± 1.99 m	19.9 ± 0.23i
	Toco 150	115.9 ± 2.23 L	19.3 ± 1.32j	4.44 ± 0.35j	138.0 ± 0.24 L	35.1 ± 2.01 L	20.0 ± 0.25 h
Bio 5	Toco 0	114.9 ± 2.21k	21.3 ± 1.42i	4.60 ± 0.41i	137.4 ± 0.19 lm	35.9 ± 2.12k	20.1 ± 0.24 h
	Toco 50	112.8 ± 2.31i	21.4 ± 1.31i	4.82 ± 0.37 h	140.9 ± 0.19k	39.1 ± 2.13j	20.3 ± 0.23 g
	Toco 100	111.0 ± 2.14 g	24.3 ± 1.42 h	5.14 ± 0.42 g	146.0 ± 0.21j	42.4 ± 2.10i	20.5 ± 0.31f
	Toco 150	108.8 ± 1.47e	27.4 ± 1.22f	5.51 ± 0.44f	151.0 ± 0.22i	46.1 ± 2.13 h	20.6 ± 0.23f
Bio 10	Toco 0	113.8 ± 1.65j	25.3 ± 1.47 g	5.58 ± 0.42ef	153.0 ± 0.18 h	52.1 ± 2.11 g	21.7 ± 0.24e
	Toco 50	109.8 ± 2.03f	33.3 ± 1.32d	5.68 ± 0.39e	158.2 ± 0.15f	57.3 ± 2.13e	21.9 ± 0.23d
	Toco 100	108.1 ± 2.14d	37.2 ± 1.33c	5.90 ± 0.39c	162.4 ± 0.19e	61.9 ± 2.14b	22.4 ± 0.22c
	Toco 150	99.7 ± 2.11b	39.9 ± 1.42b	6.47 ± 0.35a	172.8 ± 0.31b	70.2 ± 1.93a	22.8 ± 0.24b
Bio 15	Toco 0	111.9 ± 2.15 h	29.5 ± 1.22e	5.59 ± 0.40ef	154.8 ± 0.24 g	53.2 ± 1.88f	21.7 ± 0.11e
	Toco 50	108.5 ± 2.31de	33.4 ± 1.41d	5.79 ± 0.43d	163.3 ± 0.25d	58.5 ± 1.58d	22.0 ± 0.23d
	Toco 100	106.8 ± 2.14c	37.4 ± 1.32c	6.06 ± 0.44b	169.1 ± 0.21c	61.3 ± 2.06c	22.3 ± 0.14c
	Toco 150	99.0 ± 1.98a	40.3 ± 1.11a	6.49 ± 0.46a	176.4 ± 0.22a	70.0 ± 2.08a	22.9 ± 0.25a

Results are presented as means ± standard error (*n* = 3). Mean values in the same column followed by a distinct lowercase letter are significantly different according to Tukey’s HSD test (*p* ≤ 0.05)

As shown in Table 9, the foliar application of α-Toco also showed significant concentration-dependent effects on the flowering time and yield components, viz., the number of capitula plant^− 1^, diameter of the main capitulum, number of seeds capitulum^− 1^, seed yield plant^− 1^, and 1000-seed weight. Compared to the control, the 100-ppm concentration advanced flowering by 3.6% and triggered a succession of increases in yield components: by 27.1%, 10.1%, 5.7%, 15.5%, and 2.4%, respectively. Likewise, the seed yield was enhanced to 1.39 t ha^− 1^ at 100-ppm, recording an increment of 15.8% against the control (1.20 t ha^− 1^). The positive response was more prominent at the highest concentration of 150 ppm, being the strongest stimulator. The best treatment reduced the number of days to flowering by 7.8%, and the yield traits were significantly elevated by 38.4%, 20.9%, 9.8%, 27.9%, and 3.8%, respectively, as compared to the control. Interestingly, the maximum concentration (150-ppm) was the best for seed yield (1.54 t ha^− 1^) with a remarkable 28.3% improvement as compared to the control plants.

Furthermore, the synergistic effect of the biochar and α-Toco, as shown in Table 9, developed a distinct hierarchy in terms of effectiveness. The results were consistently better for the treatment combinations of either 10 or 15 t ha⁻¹ biochar with 150 ppm α-Toco. In fact, a reduction in days to 50% flowering with 15 t ha⁻¹ biochar × 150 ppm α-Toco was the highest (16.7%), and it was closely followed by 10 t ha⁻¹ biochar ×150 ppm α-Toco (16.1%). The same trend was even more pronounced for the number of capitula plant^− 1^, showing increases of 161.7% and 159.1%, respectively. Both treatments were statistically at par with each other in the case of main capitulum diameter and seed yield plant^− 1^ and were superior to the control by more than 103.4% or 102.8% and 118.1% or 118.7%, respectively. Once more, 15 t ha⁻¹ biochar × 150 ppm α-Toco also led in seeds capitulum^− 1^ (29.9% increase) and 1000-seed weight (16.8% increase) with 10 t ha⁻¹ biochar × 150 ppm α-Toco coming a close second (27.2% and 16.3%, respectively). A soil amendment of either 10-t ha^− 1^ or 15-t ha^− 1^ of biochar gave an equivalent and significant response when applied with foliar treatment with 150-ppm α-Toco (Fig. 2). These two synergistic treatments yielded the maximum recorded seed yield of 1.95 t ha^− 1^ and were statistically equivalent. This peak productivity is a very substantial 119.1% increase over the untreated control, which yielded 0.89 t ha^− 1^.

### HPLC analysis of silymarin and related constituents

The analytical data indicated a clear positive trend in the enhancement of flavonolignan composition and total silymarin content in response to biochar and α-Toco applications (Table 10). The maximum total silymarin content of 3.172% was obtained at the treatment level of 15 t ha⁻¹ biochar with 150 ppm α-Toco. Over the untreated control (Bio 0 × Toco 0), which recorded 2.230%, this value showed an increase of 42.2%. At the highest biochar level considered (15 t ha⁻¹), the application of 150 ppm α-Toco elevated total silymarin by 67.7% compared to the 100-ppm treatment (1.892%). In contrast, at 5 t ha⁻¹ biochar, increasing α-Toco from 100 ppm to 150 ppm gave 20.0% more total silymarin. On the other hand, for the individual flavonolignans, the highest-dose treatment (15-t ha⁻¹ biochar × 150 ppm α-Toco) also gave the highest concentration of Silybin A (5.0 mg g⁻¹ DW) and Silybin B (4.6 mg g⁻¹ DW). Other notable total silymarin values were 2.674% for 10-t ha⁻¹ biochar × 150 ppm α-Toco and 2.129% for 10-t ha⁻¹ biochar × 100 ppm α-Toco.

**Table 10:** Effect of the interaction between biochar application and α-tocopherol on flavonolignan composition and total silymarin content of milk thistle grown in (S_II_) 2024/25 winter season

Treatment	Flavonolignan composition (mg g⁻¹ DW)						Silymarin content (%)
Biochar (Bio; t ha^− 1^) × Tocopherol (Toco; ppm)	Silychristin	Silydianin	Silybin A	Silybin B	Isosilybin A	Isosilybin B	Total
Bio 0 × Toco 0	0.0	1.0	3.5	3.3	2.7	2.3	2.230
Bio 0 × Toco 50	0.5	0.4	1.8	1.9	3.9	7.0	1.744
Bio 0 × Toco 100	0.0	0.8	3.0	2.8	2.3	2.1	1.945
Bio 0 × Toco 150	1.4	0.4	1.7	1.6	2.0	3.0	1.515
Bio 5 × Toco 0	1.8	0.4	1.8	1.7	2.3	3.6	1.729
Bio 5 × Toco 50	0.0	1.0	3.5	3.3	2.7	2.4	2.259
Bio 5 × Toco 100	0.0	0.8	3.4	3.2	2.6	2.3	2.192
Bio 5 × Toco 150	0.0	1.1	4.1	3.8	3.2	2.8	2.631
Bio 10 × Toco 0	0.0	0.6	2.3	2.3	1.8	1.6	1.531
Bio 10 × Toco 50	0.0	0.8	2.8	2.7	2.2	1.9	1.816
Bio 10 × Toco 100	1.1	0.7	2.7	2.6	2.7	3.3	2.129
Bio 10 × Toco 150	0.0	1.1	4.1	3.9	3.2	2.8	2.674
Bio 15 × Toco 0	0.1	1.0	3.6	3.4	2.8	2.5	2.346
Bio 15 × Toco 50	0.0	0.6	2.7	2.6	2.1	1.8	1.745
Bio 15 × Toco 100	0.0	0.8	2.9	2.8	2.2	2.0	1.892
Bio 15 × Toco 150	0.0	1.4	5.0	4.6	3.8	3.2	3.172

Data are presented as a percentage of total silymarin (calculated as silybin). $\text{Silymarin content}\left(\%\right)\:\frac{\mathbf{A}\mathbf{r}\mathbf{e}\mathbf{a}\:\mathrm{T}\mathrm{e}\mathrm{s}\mathrm{t}}{\mathbf{A}\mathbf{r}\mathbf{e}\mathbf{a}\:\mathrm{S}\mathrm{t}\mathrm{d}}\:\times\:\:\frac{\:\mathbf{C}\mathbf{o}\mathbf{n}\mathbf{c}.\:\:\mathrm{S}\mathrm{t}\mathrm{d}}{\:\mathbf{C}\mathbf{o}\mathbf{n}\mathbf{c}.\:\:\mathrm{T}\mathrm{e}\mathrm{s}\mathrm{t}}\times\:\mathbf{P}\mathbf{o}\mathbf{t}\mathbf{e}\mathbf{n}\mathbf{c}\mathbf{y}$

### Leaf and stem anatomical structures

**Fig. 3:**
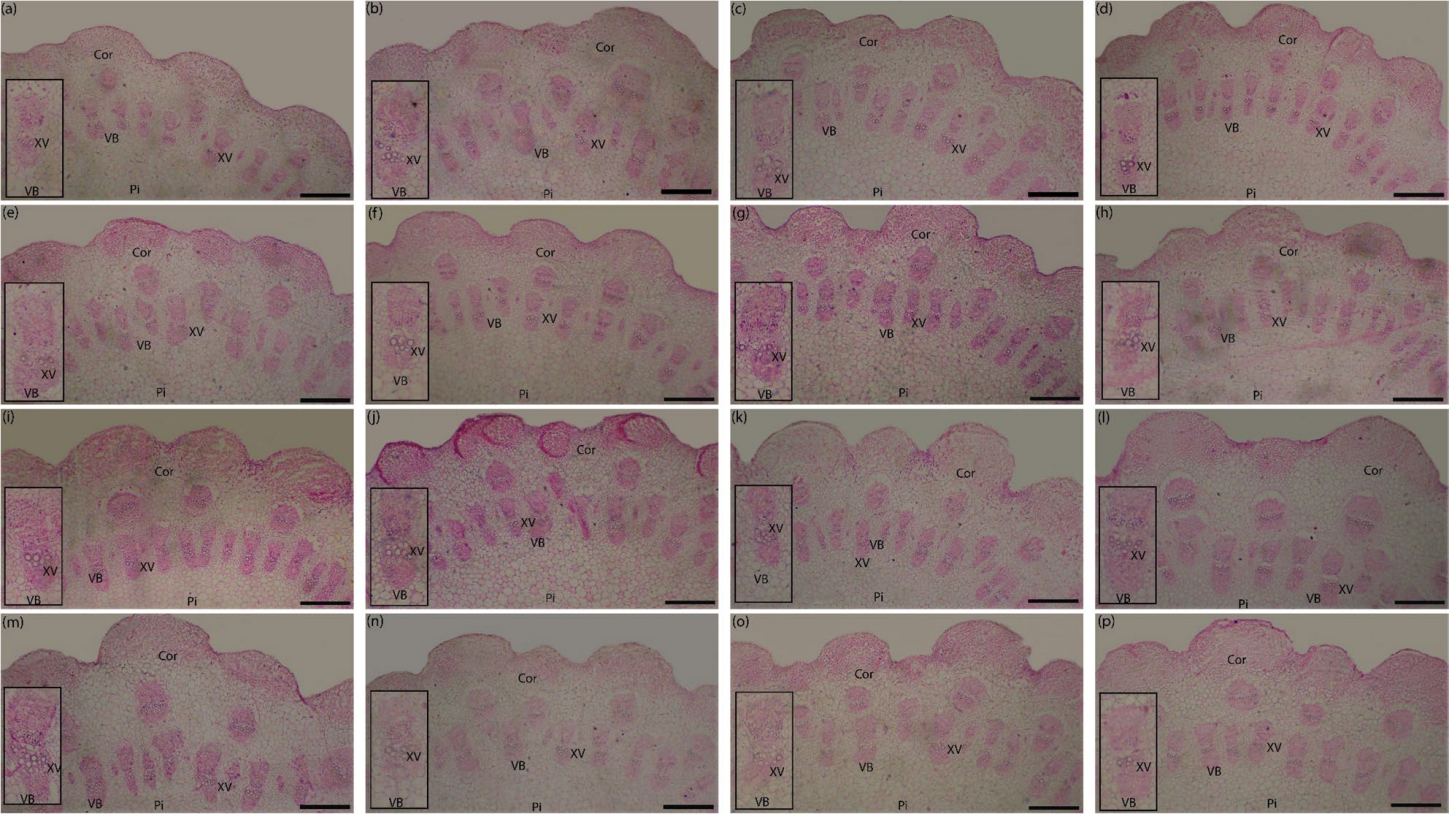
Effect of biochar application and α-tocopherol, and their interaction on transections of stem of milk thistle grown in (SI) 2023/24 winter season. Cortex (Cor), xylem vessels (XV), vascular bundles (VB), and pith (Pi). Bars = 500 μm. Bio 0 × Toco 0 (**a**), Bio 0 × Toco 50 (**b**), Bio 0 × Toco 100 (**c**), Bio 0 × Toco 150 (**d**), Bio 5 × Toco 0 (**e**), Bio 5 × Toco 50 (**f**), Bio 5 × Toco 100 (**g**), Bio 5 × Toco 150 (**h**), Bio 10 × Toco 0 (**i**), Bio 10 × Toco 50 (**j**), Bio 10 × Toco 100 (**k**), Bio 10 × Toco 150 (**l**), Bio 15 × Toco 0 (**m**), Bio 15 × Toco 50 (**n**), Bio 15 × Toco 100 (**o**), Bio 15 × Toco 150 (**p**)

**Fig. 4:**
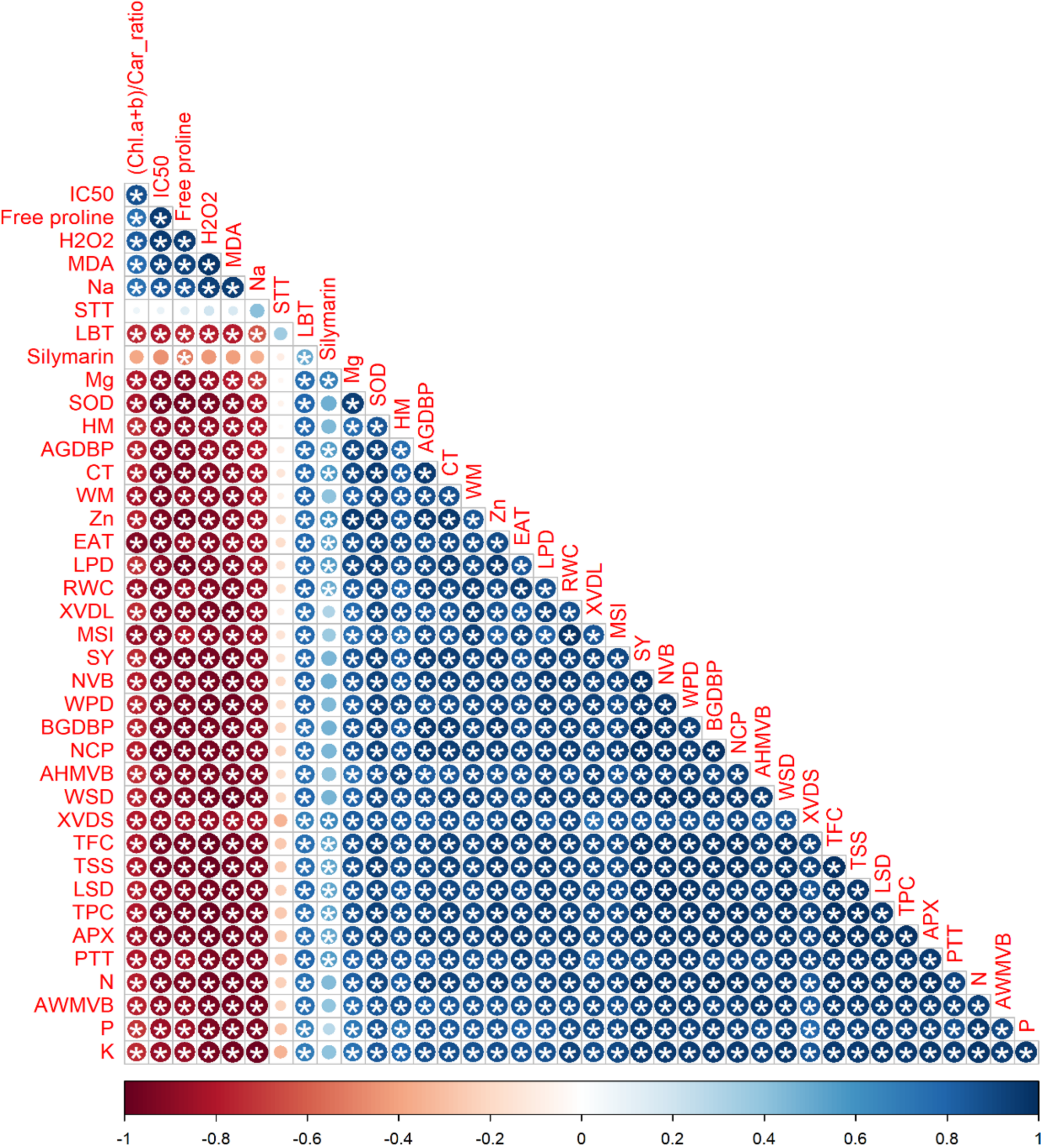
Analysis of Pearson’s correlation among all studied parameters and the colors represent the variations in the obtained data. * indicates significant at *p*-adjusted value (“Tukey’s HSD” method) ≤ 0.05. Sodium (Na), malondialdehyde (MDA), hydrogen peroxide (H_2_O_2_), half-maximal inhibitory levels (IC_50_), total chlorophyll/ total carotenoid (Chl *a* + *b*/Car ratio), potassium (K), phosphorus (P), average of width of midvein vascular bundle (AWMVB), nitrogen (N), palisade tissue thickness (PTT), ascorbate peroxidase (APX), total phenolic content (TPC), total soluble sugars (TSS), total flavonoid content (TFC), number of capitula plant^− 1^ (NCP), average of height of midvein vascular bundle (AHMVB), below ground dry biomass plant^− 1^ (BGDBP), seed yield ha^− 1^ (SY), membrane stability index (MSI), xylem vessel diameter (XVD), relative water content (RWC), zinc (Zn), endogenous alpha-tocopherol (EAT), width of midvein (WM), above ground dry biomass plant^− 1^ (AGDBP), superoxide dismutase (SOD), height of midvein (HM), magnesium (Mg), leaf blade thickness (LBT), spongy tissue thickness (STT), length stem dimensions (LSD), width stem dimensions (WPD), length pith dimension (LPD), width pith dimension (WPD), cortex thickness (CT), number of vascular bundles (NVB), and stem xylem vessel diameter (XVDS)

According to the results shown in Tables 11 and 12; Figs. 3 and 4, biochar soil application had a significant impact on leaf tissue architecture. A gradual increase, tending toward positive linearity, in the majority of leaf and stem anatomical parameters was found as increasing rates of biochar from 0 to 15 t ha⁻¹ were applied. Particularly, the most robust anatomical profile was achieved with the application of the highest dose (15 t ha⁻¹) when compared to the salt-stressed control (0 t ha⁻¹) milk thistle plants. Plants amended with 15 t ha⁻¹ of biochar showed remarkable increases in leaf anatomical parameters i.e., width of midvein (WM) and height of midvein (HM) (35.0% and 40.0%, respectively). In the same way, the vascular system dimensions increased significantly; the midvein vascular bundle’s average height (AHMVB) and width (AWMVB) increased by 41.1 and 45.4%, respectively, over the non-amended plants. Leaf blade thickness (LBT) and palisade tissue thickness (PTT) were also improved significantly at the highest biochar level with increases of 7.6 and 39.2%, respectively. By contrast, spongy tissue thickness (STT) demonstrated the reverse pattern, reducing by 7.6% in response to the same treatment. The treatment also significantly increased the xylem vessel diameter (XVDL) by 28.6% compared to the control. A similar treatment also resulted in a significant increase in all stem anatomical parameters in comparison to the control. More in detail, it promoted increases in the following: stem length dimensions (LSD) by 44.4%, stem width dimensions (WSD) by 45.5%, pith length dimensions (LPD) by 50.8%, pith width dimensions (WPD) by 51.6%, cortex thickness (CT) by 40.6%, number of vascular bundle (NVB) by 40.4%, and xylem vessel diameter (XVDS) by 25.8%.

**Table 11:** Effect of biochar application and α-tocopherol, and their interaction on leaf anatomical structures of milk thistle grown in (S_I_) 2023/24 winter season

Treatments		WM	HM	AHMVB	AWMVB	LBT	PTT	STT	XVD
		µm							
Biochar (Bio) t ha^− 1^									
0		2320.8 ± 4.12d	2231.3 ± 6.21d	562.5 ± 2.54d	293.8 ± 1.15d	495.8 ± 4.12b	157.5 ± 1.11c	285.8 ± 2.11a	32.9 ± 1.11d
5		2393.8 ± 4.15c	2477.1 ± 6.58c	643.8 ± 2.42c	352.1 ± 2.12c	496.7 ± 3.21b	194.2 ± 1.23b	252.5 ± 1.58c	33.9 ± 0.89c
10		3066.7 ± 6.32b	2702.1 ± 8.32b	735.4 ± 3.21b	397.9 ± 2.13b	532.5 ± 3.25a	212.5 ± 2.35a	270.0 ± 3.35b	40.7 ± 0.1.10b
15		3133.3 ± 5.21a	3122.9 ± 9.25a	793.8 ± 1.87a	427.1 ± 2.11a	533.3 ± 4.23a	219.2 ± 3.35a	264.2 ± 2.58b	42.3 ± 0.99a
Tocopherol (Toco) ppm									
0		2364.6 ± 4.12d	2262.5 ± 6.32d	629.2 ± 2.58c	335.4 ± 2.32c	481.7 ± 3.25c	175.0 ± 2.32d	256.7 ± 3.11c	34.9 ± 0.97d
50		2629.2 ± 3.25c	2662.5 ± 4.25c	685.4 ± 2.32b	370.8 ± 2.12b	524.2 ± 3.26ab	187.5 ± 1.25c	285.8 ± 3.24a	36.8 ± 0.69c
100		2904.2 ± 2.65b	2750.0 ± 4.36b	687.5 ± 2.35b	366.7 ± 2.11b	520.8 ± 3.62b	205.0 ± 1.33b	264.2 ± 2.35b	38.3 ± 0.1.11b
150		3016.7 ± 4.25a	2858.3 ± 4.36a	733.3 ± 1.36a	397.9 ± 2.02a	531.7 ± 3.25a	215.8 ± 1.45a	265.8 ± 1.78b	39.9 ± 0.1.12a
Bio × Toco									
Bio 0	Toco 0	1975.0 ± 0.98 L	1766.7 ± 2.23k	500.0 ± 1.89j	258.3 ± 1.89i	463.3 ± 2.01 h	140.0 ± 1.65i	270.0 ± 2.10 cd	29.0 ± 0.75i
	Toco 50	2125.0 ± 0.65k	2475.0 ± 2.36gh	558.3 ± 2.01i	300.0 ± 1.11 g-i	520.0 ± 1.11c-f	156.7 ± 1.75 h	313.3 ± 2.22a	30.7 ± 0.65 h
	Toco 100	2566.7 ± 1.12 h	2400.0 ± 1.56 h	591.7 ± 2.11hi	291.7 ± 1.12hi	510.0 ± 1.11e-g	163.3 ± 0.98 h	290.0 ± 1.11b	35.0 ± 0.32 g
	Toco 150	2616.7 ± 2.10 h	2283.3 ± 1.96i	600.0 ± 1.65 h	325.0 ± 1.23f-h	490.0 ± 1.18 g	170.0 ± 1.25gh	270.0 ± 2.32 cd	37.0 ± 0.45f
Bio 5	Toco 0	2125.0 ± 1.58k	2208.3 ± 4.25j	600.0 ± 1.87 h	316.7 ± 1.21f-h	443.3 ± 2.11i	163.3 ± 1.22 h	230.0 ± 1.14f	31.7 ± 0.63 h
	Toco 50	2275.0 ± 1.89j	2550.0 ± 4.23 fg	650.0 ± 1.89 g	341.7 ± 1.11e-g	510.0 ± 0.0e-g	183.3 ± 1.11 fg	276.7 ± 1.11b-d	35.0 ± 0.42 g
	Toco 100	2450.0 ± 2.01i	2433.3 ± 4.21 h	608.3 ± 2.02 h	358.3 ± 1.12d-f	513.3 ± 1.32ef	210.0 ± 2.11c-e	250.0 ± 1.11e	34.0 ± 0.32 g
	Toco 150	2725.0 ± 0.45 g	2716.7 ± 3.21e	716.7 ± 2.25ef	391.7 ± 1.13 cd	520.0 ± 2.03c-f	220.0 ± 1.11b-d	253.3 ± 2.25e	35.0 ± 0.47 g
Bio 10	Toco 0	2450.0 ± 0.71i	2475.0 ± 2.21gh	691.7 ± 2.25f	375.0 ± 1.18c-e	503.3 ± 1.87 fg	193.3 ± 0.89ef	263.3 ± 0.98de	39.0 ± 0.63e
	Toco 50	3125.0 ± 0.63d	2725.0 ± 3.21e	758.3 ± 2.12 cd	400.0 ± 1.89b-d	536.7 ± 1.89b-d	200.0 ± 1.23e	283.3 ± 2.25bc	40.3 ± 0.35de
	Toco 100	3375.0 ± 0.66a	2775.0 ± 1.21de	750.0 ± 1.11c-e	400.0 ± 1.58b-d	540.0 ± 1.76bc	226.7 ± 1.36bc	263.3 ± 1.25de	41.0 ± 0.42 cd
	Toco 150	3316.7 ± 0.85b	2833.3 ± 1.11 cd	741.7 ± 1.18c-e	416.7 ± 1.87a-c	550.0 ± 1.65ab	230.0 ± 1.89ab	270.0 ± 1.30 cd	42.3 ± 0.44bc
Bio 15	Toco 0	2908.3 ± 1.25f	2600.0 ± 1.01f	725.0 ± 1.18d-f	391.7 ± 1.85 cd	516.7 ± 1.75d-f	203.3 ± 1.99de	263.3 ± 1.42de	40.0 ± 0.52de
	Toco 50	2991.7 ± 1.25e	2900.0 ± 1.13c	775.0 ± 1.25bc	441.7 ± 1.36ab	530.0 ± 1.63b-e	210.0 ± 1.11c-e	270.0 ± 1.20 cd	41.0 ± 1.01 cd
	Toco 100	3225.0 ± 2.01c	3391.7 ± 1.15b	800.0 ± 2.12b	416.7 ± 1.45a-c	520.0 ± 1.54c-f	220.0 ± 1.23b-d	253.3 ± 1.21e	43.0 ± 0.99b
	Toco 150	3408.3 ± 2.11a	3600.0 ± 1.11a	875.0 ± 2.13a	458.3 ± 2.02a	566.7 ± 1.35a	243.3 ± 2.11a	270.0 ± 1.11 cd	45.3 ± 0.75a

Results are presented as means ± standard error (*n*=3). Mean values in the same column followed by a distinct lowercase letter are significantly different according to Tukey's HSD test (*p* ≤ 0.05)

*WM *Width of midvein, *HM *height of midvein, *AHMVB *average of height of midvein vascular bundle, *AWMVB *average of width of midvein vascular bundle, *LBT *leaf blade thickness, *PTT *palisade tissue thickness, *STT *spongy tissue thickness, and *XVD *xylem vessel diameter

**Table 12:** Effect of biochar application and α-tocopherol, and their interaction on stem anatomical structures of milk thistle grown in (S_I_) 2023/24 winter season

Treatments		LSD	WSD	LPD	WPD	CT	NVB	XVDS
		µm						
Biochar (Bio) t ha^− 1^								
0		5410.4 ± 5.23c	4747.9 ± 8.32d	3475.0 ± 9.62d	2908.3 ± 7.62d	320.0 ± 3.14c	61.9 ± 0.31c	22.1 ± 0.23c
5		6481.3 ± 8.36b	5520.8 ± 5.69c	3791.7 ± 9.11c	3385.4 ± 9.52c	358.3 ± 3.23b	70.3 ± 0.22b	25.4 ± 0.56b
10		7666.7 ± 9.32a	6625.0 ± 7.21b	4729.2 ± 8.21b	4156.3 ± 7.11b	440.8 ± 2.14a	86.1 ± 0.72a	26.3 ± 0.42b
15		7812.5 ± 7.65a	6906.3 ± 6.92a	5239.6 ± 8.61a	4410.4 ± 4.61a	450.0 ± 1.41a	86.9 ± 0.34a	27.8 ± 0.35a
Tocopherol (Toco) ppm								
0		6262.5 ± 9.32d	5456.3 ± 7.25d	3929.2 ± 7.66c	3312.5 ± 8.36d	348.3 ± 2.95d	69.8 ± 0.23d	23.4 ± 0.34c
50		6531.3 ± 7.32c	5737.5 ± 8.62c	4004.2 ± 9.16c	3562.5 ± 9.11c	375.8 ± 3.32c	73.2 ± 0.31c	24.2 ± 0.45c
100		6954.2 ± 6.32b	6197.9 ± 7.32b	4456.3 ± 9.12b	3816.7 ± 8.36b	411.7 ± 2.26b	78.8 ± 0.33b	26.3 ± 0.36b
150		7622.9 ± 8.21a	6408.3 ± 7.36a	4845.8 ± 8.51a	4168.8 ± 9.01a	433.3 ± 1.19a	83.4 ± 0.42a	27.7 ± 0.47a
Bio × Toco								
Bio 0	Toco 0	4900.0 ± 7.25 h	4283.3 ± 9.36i	3083.3 ± 9.11i	2375.0 ± 5.12 g	310.0 ± 2.13d	55.7 ± 0.61i	21.0 ± 0.71f
	Toco 50	5250.0 ± 5.36gh	4658.3 ± 9.21hi	3350.0 ± 5.91 h	3000.0 ± 5.11ef	326.7 ± 2.25d	61.0 ± 0.71 h	21.7 ± 0.65ef
	Toco 100	5616.7 ± 8.69 fg	5000.0 ± 10.3gh	3750.0 ± 8.15f	3000.0 ± 8.12ef	316.7 ± 1.96d	66.0 ± 0.21 g	23.0 ± 0.67d-f
	Toco 150	5875.0 ± 9.36f	5050.0 ± 9.81gh	3716.7 ± 8.91 fg	3258.3 ± 4.12e	326.7 ± 1.65d	65.0 ± 0.32 g	22.7 ± 0.64d-f
Bio 5	Toco 0	5683.3 ± 8.36 fg	4791.7 ± 8.32 h	3508.3 ± 7.58gh	2916.7 ± 3.22f	323.3 ± 2.25d	63.3 ± 0.61gh	24.3 ± 0.52d-f
	Toco 50	6000.0 ± 9.32f	5375.0 ± 8.41 g	3625.0 ± 6.36 fg	3125.0 ± 4.52ef	366.7 ± 1.25c	65.0 ± 0.36 g	24.0 ± 0.71d-f
	Toco 100	7000.0 ± 9.21e	5875.0 ± 8.51f	4033.3 ± 8.32e	3625.0 ± 5.32d	376.7 ± 3.21c	75.0 ± 0.45f	25.0 ± 0.66cde
	Toco 150	7241.7 ± 6.35de	6041.7 ± 8.42f	4000.0 ± 7.65e	3875.0 ± 6.62 cd	366.7 ± 1.69c	77.7 ± 0.36ef	28.3 ± 0.71a-c
Bio 10	Toco 0	7250.0 ± 7.21de	6166.7 ± 8.51ef	4250.0 ± 8.36d	3916.7 ± 3.32 cd	370.0 ± 1.65c	78.7 ± 0.45de	22.3 ± 0.72ef
	Toco 50	7250.0 ± 8.32de	6291.7 ± 9.41d-f	4166.7 ± 7.36de	4000.0 ± 4.33c	383.3 ± 1.09c	81.7 ± 0.32 cd	25.0 ± 0.43c-e
	Toco 100	7750.0 ± 9.32c	7000.0 ± 8.41bc	5083.3 ± 7.36c	4083.3 ± 5.45c	500.0 ± 2.02a	90.0 ± 0.32b	28.3 ± 0.45a-c
	Toco 150	8416.7 ± 7.36b	7041.7 ± 9.14b	5416.7 ± 4.32b	4625.0 ± 4.95ab	510.0 ± 1.23a	94.0 ± 0.42a	29.7 ± 0.66a
Bio 15	Toco 0	7216.7 ± 8.36de	6583.3 ± 7.41c-e	4875.0 ± 3.91c	4041.7 ± 5.31c	390.0 ± 2.11c	81.7 ± 0.35 cd	26.0 ± 0.45b-d
	Toco 50	7625.0 ± 6.32 cd	6625.0 ± 6.32b-d	4875.0 ± 4.19c	4125.0 ± 5.62c	426.7 ± 1.96b	85.0 ± 0.45c	26.0 ± 0.63b-d
	Toco 100	7450.0 ± 4.21cde	6916.7 ± 7.42bc	4958.3 ± 5.21c	4558.3 ± 1.92b	453.3 ± 2.23b	84.0 ± 0.25c	29.0 ± 0.66ab
	Toco 150	8958.3 ± 5.36a	7500.0 ± 8.45a	6250.0 ± 4.52a	4916.7 ± 2.92a	530.0 ± 1.98a	97.0 ± 0.32a	30.0 ± 0.73a

Results are presented as means ± standard error (*n*=3). Mean values in the same column followed by a distinct lowercase letter are significantly different according to Tukey's HSD test (*p* ≤ 0.05)

*LSD* length of stem dimensions, *WSD* width of stem dimensions, *LPD* length of pith dimension, *WPD* width of pith dimension, *CT* cortex thickness, *NVB* number of vascular bundle, and *XVDS *xylem vessel diameter

Similarly, foliar application of α-Toco enhanced all anatomical leaf and stem parameters in milk thistle as compared to non-sprayed (control) plants (Table 11; Fig. 3). Our observations indicate that mid-to-high levels of α-Toco (100 or 150 ppm) most often increased parameters. These treatments raised WM by 22.8 or 27.6%, HM by 21.6 or 26.3%, AHMVB by 9.3 or 16.5%, AWMVB by 9.3 or 18.6%, LBT by 8.1 or 10.4%, PTT by 17.1 or 23.3%, and XVDL by 9.7 or 14.3%, compared with control, respectively. In contrast, STT exhibited only marginal improvements, with 2.9% and 3.5% at 100 and 150 ppm, respectively. Additionally, foliar application of α-Toco at 150 ppm significantly enhanced all the stem anatomical characteristics of the plant compared to the control, with increases in LSD, WSD, LPD, WPD, CT, NVB, and XVDS by 21.7%, 17.4%, 23.3%, 25.8%, 24.4%, 19.5%, and 18.4%, respectively.

A synergistic and highly significant interaction of biochar and α-Toco was observed regarding the measure of all anatomical traits. The most enhanced leaf anatomical characteristics were consistently recorded with a combined maximum dose application of both biochar (15 t ha⁻¹) and α-Toco (150 ppm). When compared with this specific combination to that of absolute control (0 t ha⁻¹ biochar and 0 ppm α-Toco), these maximum values represent a colossal increase: 72.6% for WM, 103.8% for HM, 75.0% for AHMVB, 77.4% for AWMVB, 22.3% for LBT, 73.8% for PTT, and 56.2% for XVDL. On the contrary, STT displayed a unique non-linear pattern, exhibiting an increase to its maximum of 16.0% (313.3 μm) with the combination of 0 t ha⁻¹ biochar and 50 ppm α-Toco. In comparison, the highest combined treatment (15 t ha⁻¹ biochar × 150 ppm α-Toco) recorded an STT figure identical to that of the control (270.0 μm). Furthermore, the dual application (15 t ha⁻¹ × 150 ppm) resulted in maximum effectiveness. Increases of about  82.8% in LSD 75.1% in WSD, 102.7% in LPD, 107.0% in WPD, 71.0% in CT, 74.1% in NVB, and 42.9% in XVDS were obtained over the lowest treatment (0 × 0). 

### Relations

**Fig. 5:**
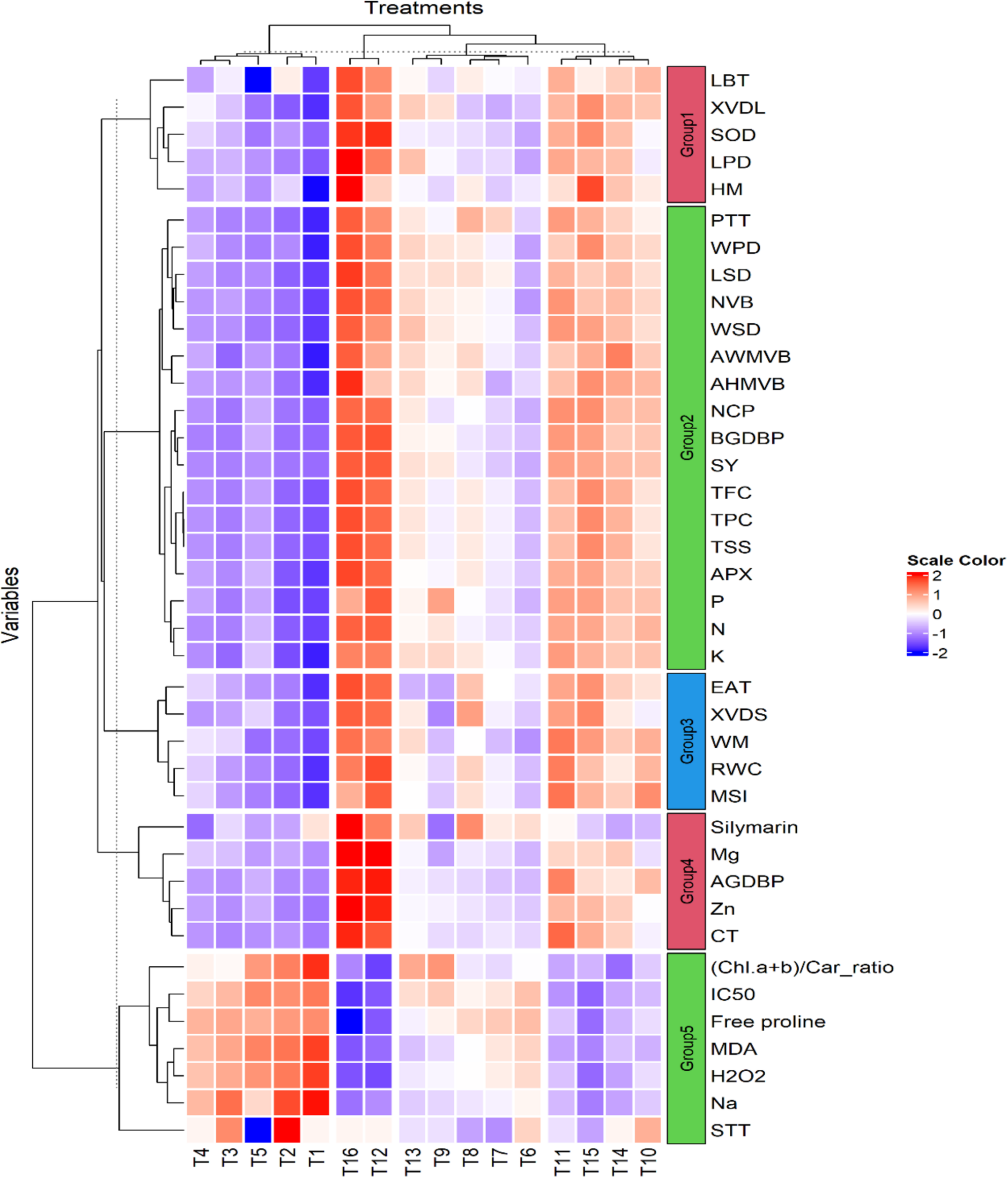
Heat map analysis of hierarchical clustering among all studied parameters and treatments and scale color representing Z-score values of each parameter data. Sodium (Na), malondialdehyde (MDA), hydrogen peroxide (H_2_O_2_), half-maximal inhibitory levels (IC_50_), total chlorophyll/ total carotenoid (Chl *a* + *b*/Car ratio), potassium (K), phosphorus (P), average of width of midvein vascular bundle (AWMVB), nitrogen (N), palisade tissue thickness (PTT), ascorbate peroxidase (APX), total phenolic content (TPC), total soluble sugars (TSS), total flavonoid content (TFC), number of capitula plant^− 1^ (NCP), average of height of midvein vascular bundle (AHMVB), below ground dry biomass plant^− 1^ (BGDBP), seed yield ha^− 1^ (SY), membrane stability index (MSI), xylem vessel diameter (XVD), relative water content (RWC), zinc (Zn), endogenous alpha-tocopherol (EAT), width of midvein (WM), above ground dry biomass plant^− 1^ (AGDBP), superoxide dismutase (SOD), height of midvein (HM), magnesium (Mg), leaf blade thickness (LBT), spongy tissue thickness (STT), xylem vessel dimension leaf (XVDL). length stem dimensions (LSD), width stem dimensions (WPD), length pith dimension (LPD), width pith dimension (WPD), cortex thickness (CT), number of vascular bundles (NVB), and stem xylem vessel diameter (XVDS). Bio 0 × Toco 0 (T1), Bio 0 × Toco 50 (T2), Bio 0 × Toco 100 (T3), Bio 0 × Toco 150 (T4), Bio 5 × Toco 0 (T5), Bio 5 × Toco 50 (T6), Bio 5 × Toco 100 (T7), Bio 5 × Toco 150 (T8), Bio 10 × Toco 0 (T9), Bio 10 × Toco 50 (T10), Bio 10 × Toco 100 (T11), Bio 10 × Toco 150 (T12), Bio 15 × Toco 0 (T13), Bio 15 × Toco 50 (T14), Bio 15 × Toco 100 (T15), Bio 15 × Toco 150 (T16)

The average data of 32 studied variables were used to detect the values of correlation and significant generated among these parameters by performing a Pearson’s correlation analysis Fig. 5. The studied parameters including physio-biochemical, growth, yield traits that influenced by the application of soil application of mango-residue biochar amendments (0; control, 5, 10, and 15 t ha^− 1^) and foliar spraying with α-Toco (0; non-spray, 50,100, and 150 ppm) to milk thistle plants under saline conditions. We have detected a positive correlation (significant at p-adjusted value ≤ 0.05) among selected growth parameters of milk thistle plants including above ground dry biomass plant^− 1^, below ground dry biomass plant^− 1^, and yield traits including number of capitula plant^− 1^, seed yield ha^− 1^, and silymarin content with HM, activities of SOD, APX, contents of K, P, N, Zn, TSS, and TFC, as well as the levels of MSI, and anatomical traits including XVDL, AHMVB, AWMVB, WSD, XVDS, PT, NVB, CT, LPD, WSD, LSD, PTT, LBT, HM, and WM. Moreover. Na, H_2_O_2_, IC_50_, free proline, and MDA levels were negatively (significant at adjusted *p* < 0.05) correlated with the above-mentioned parameters.

**Fig. 6:**
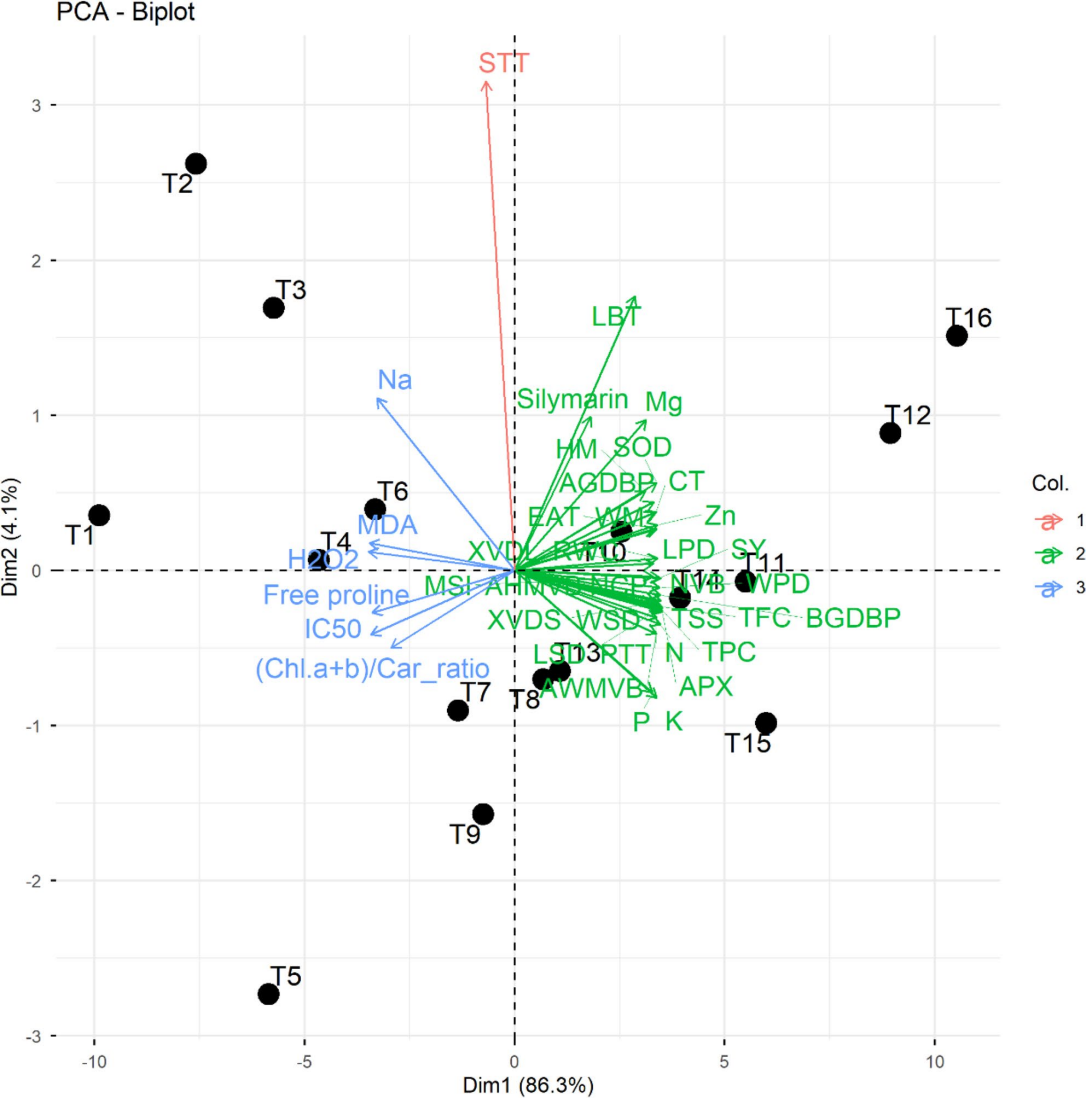
Bi-plot graph of studied parameters and treatments, showing the first two principal component analysis (PCA) dimensions (Dim1 and Dim2). Sodium (Na), malondialdehyde (MDA), hydrogen peroxide (H_2_O_2_), half-maximal inhibitory levels (IC_50_), total chlorophyll/ total carotenoid (Chl *a* + *b*/Car ratio), potassium (K), phosphorus (P), average of width of midvein vascular bundle (AWMVB), nitrogen (N), palisade tissue thickness (PTT), ascorbate peroxidase (APX), total phenolic content (TPC), total soluble sugars (TSS), total flavonoid content (TFC), number of capitula plant^− 1^ (NCP), average of height of midvein vascular bundle (AHMVB), below ground dry biomass plant^− 1^ (BGDBP), seed yield ha^− 1^ (SY), membrane stability index (MSI), xylem vessel diameter (XVD), relative water content (RWC), zinc (Zn), endogenous alpha-tocopherol (EAT), width of midvein (WM), above ground dry biomass plant^− 1^ (AGDBP), superoxide dismutase (SOD), height of midvein (HM), magnesium (Mg), leaf blade thickness (LBT), spongy tissue thickness (STT), xylem vessel dimension leaf (XVDL). length stem dimensions (LSD), width stem dimensions (WPD), length pith dimension (LPD), width pith dimension (WPD), cortex thickness (CT), number of vascular bundles (NVB), and stem xylem vessel diameter (XVDS). Bio 0 × Toco 0 (T1), Bio 0 × Toco 50 (T2), Bio 0 × Toco 100 (T3), Bio 0 × Toco 150 (T4), Bio 5 × Toco 0 (T5), Bio 5 × Toco 50 (T6), Bio 5 × Toco 100 (T7), Bio 5 × Toco 150 (T8), Bio 10 × Toco 0 (T9), Bio 10 × Toco 50 (T10), Bio 10 × Toco 100 (T11), Bio 10 × Toco 150 (T12), Bio 15 × Toco 0 (T13), Bio 15 × Toco 50 (T14), Bio 15 × Toco 100 (T15), Bio 15 × Toco 150 (T16)

A heat map with hierarchical analysis was performed and visualized to reveal the interactive connection between the studied treatments application of biochar amendments and α-Toco to milk thistle plants under saline conditions, as presented in Fig. 6. The studied treatments (X-axis) were separated using the hierarchical cluster analysis into four main groups. T4, T3, T5, T2, and T1 were separated in the first main group, while the second group included T14 and T12. The third group was including T13, T9, T8, T7, and T6. While the fourth group included T15, T1, T14, and T10. Moreover, the Y axis (selected parameters) were divided into four main groups (Group1, Group2, Group3 and Group4). Group1 included LBT, Mg, silymarin contents, while the group2 included PTT, WPD, LSD, NVB, WSD, AWMVB, AHMVB, EAT, WM, RWC, MSI, above ground dry biomass plant^− 1^(AGDBP), SOD, Zn, number of capitula plant^− 1^ (NCP), below-ground dry biomass plant^− 1^ (BGDBP), seed yield ha^− 1^ (SY), TFC, TPC, TSS, APX, P, N, and K. The group 3 included EAT, XVDS, WM, RWC, MSI. The group 4 included Zn, Mg, silymarin contents, CT, and AGDBP, while the group 5 included other remain parameters including STT, Na, H_2_O_2_, MDA, free proline, IC_50_, and ratio of Chl a + b/Car. These results highlighted that application of biochar application with α-Toco can effectively improve milk thistle yield and silymarin yield, antioxidant enzyme activities, and several physiological indices.

Besides the above, principal component analysis (PCA)-biplot was performed to explore the high variations resulted by application of biochar amendments and α-Toco to milk thistle plants under saline conditions. Dim 1 and Dim 2 (PCA-diminution 1 and 2, respectively) explored 85% and 5% variability of data, respectively (Fig. 6). The elevated variability between T1 and T16 in a hand and between T1 and T12 indicated the high variability in plant growth of milk thistle plants by application of biochar amendments and α-Toco. The application of biochar amendments and α-Toco to milk thistle plants under salinity stress improved silymarin yield, plant dry weight, antioxidant enzyme activities, contents of nutrient elements, and anatomical treats, while decreased the oxidative stress including MDA, Na, H_2_O_2_, and IC_50_. Therefore, application of biochar amendments and α-Toco has a distinct role in improving the yield, growth performance and physio-biochemical traits in milk thistle plants under salinity stress conditions.

## Discussion

Climate change threatens global agricultural sustainability very much by increasing the occurrence and magnitude of abiotic stresses, for example, soil salinity [4]. The matter is being more aggravated in arid and semi-arid lands where alterations in precipitation patterns and greater evaporation will lead to the deposition of soluble salts right into the root zone, thereby challenging the soil structure, diminishing nutritional availability, and putting stress on the plants through osmosis and ion toxicity [5, 54], thus viciously curbing any kind of crop growth and productivity. Amongst alternate strategies adopted for effective prevention, biochar application presented an increasingly encouraging soil amendment to mitigate such a consequence of salinity due to its property of altering the physical, chemical, and biological characteristics of soil [55, 56]. In this respect, thorough analyses have been made of biochar and soil properties before application of biochar and after its addition. The biochar analyzed showed pH 7.31, ECe 2.98 dS m^− 1^, P 0.26%, K 0.74%, and N 1.97% (Table 3). Initial analysis of the soil before the plantation showed a very high salinity rate (ECe = 8.55 dS m^− 1^), which usually reflects pronounced salt-affected conditions (Table 1). Such high values of all these parameters evidently reflect the ordinary problems associated with saline soil development under the ever-changing climatic conditions [57]. Surprisingly, after the soil was amended with biochar at the rate of 10 t ha^− 1^ (Table 2), a radical improvement in salinity indices was witnessed with a staggering 28.2% drop in soil ECe to 6.14 dS m^− 1^. This reduction is primarily attributed to biochar’s high adsorption capacity and cation exchange capacity (CEC), which effectively sequestered soluble Na^+^ and Cl^−^ ions [58]. The concentrations of soluble magnesium, potassium, sulfate, and other nutrients also changed, indicating an improvement in the ion balance and nutrient availability of the soil. Collectively, these findings indicate that biochar has the potential to alter the chemical environment of salt-impacted soils and thus promote plant growth and increased protection from climate-driven abiotic stresses [58].

The drastic improvement in the chemical properties of soil made a more favorable environment in the root zone, which converted into higher growth and biomass production of milk thistle (Table 4). Biochar’s positive effect on overall plant vigor under saline conditions observed in our experiments aligns with very recent reports of Derbali et al. [59] on quinoa and Ali et al. [60] on sunflower. The prominent growth stimulation can be ascribed to the versatility of biochar in mitigating salinity stresses. Biochar reduces the soil osmotic stress confirmed *via* analysis, by either reducing the EC of the soil or by adsorbing excess Na^+^ and cl^−^ ions and accordingly makes it easy for a plant to uptake water and essential nutrients [61]. Also, an improved water status is very important in cell turgor maintenance, which is necessary for cell enlargement and division that ultimately produce the increases noticed in plant height, number of branches, and total biomass accumulation [62–64]. As a result, the larger root system was more effective in resource acquisition [65], which accounted for its greater vegetative growth.

The second strategy for reducing salt stress, which works in tandem with soil amendments, involves the foliar application of antioxidants to prime internal defense mechanisms of plants [66]. This promotes a fast absorption from the stomata in the leaves and a rapid translocation to different plant organs, allowing a rapid reaction to cellular stress [67]. In our study, the decrease in milk thistle growth under control conditions (non-spray) could be ascribed to the combined effect of salt-mediated osmotic and oxidative stress. The oxidative damage was antagonized by the exogenous application of α-Toco [68]. Among the lipid-soluble antioxidants, α-Toco is effective at inserting itself into the cellular membranes, where it protects membrane lipids from peroxidation by ROS [69]. In addition, by assuring removal from these toxic ROS, it safeguards the structural integrity of important photosynthesis apparatus and thereby helps to maintain overall metabolism, reflecting in the vigorous growth and significant biomass accumulation of treated plants [22].

Photosynthetic pigments are the metabolic substrate for carbon uptake and biomass accumulation [70]. However, under saline conditions, the toxic ion accumulation leads to disruption in the ultrastructure of chloroplast and leads to oxidative stress whereby pigment biosynthesis is distorted and chlorophyll degradation occurs more rapidly [71, 72]. The consequence is the significant reduction in Chl *a*, *b*, and Car, which was seen in untreated plants (Table 5). However, the increase in photosynthetic pigments is the solid evidence for the stress-alleviating capability of biochar, which might be through the enhancement of two major pathways [73]. Foliar application of α-Toco, a major chloroplast antioxidant, alleviates salinity stress by scavenging ROS and suppressing the propagation of thylakoid lipid peroxidation [25]. This preserves the photosynthetic pigments and also enhances the MSI, thus enabling better osmotic regulation and RWC for the plant. Additionally, synergistic enhancement of pigmentation results from a concerted combination of two actions. Biochar alleviated ionic antagonism in the soil and thus increased the availability of Mg²⁺, the essential element of chlorophyll [74]. At the same time, foliar α-Toco also played an important role as an intracellular antioxidant that prevented the enzymatic apparatus from being damaged by ROS [75, 76].

Salt stress usually presents a two-pronged difficulty for plants: it leads to a perturbation in ion homeostasis via an excessive accumulation of Na^⁺^ that antagonistically impedes the uptake of other essential nutrients, in particular, K^⁺^, and causes high oxidative stress at the same time [77]. Our results suggest that the amendment with biochar can effectively nullify such adverse effects through multiple mechanisms operating in concert. Biochar amendment gave the first soil ameliorative effect through its high CEC emerging from its vast specific surface area that probably restricted Na^+^ bioavailability in the soil solution through adsorption [78]. Biochar was a nutrient reservoir, though, releasing K^+^, Mg^2+^, and Zn^2+^ cations slowly, which displaced Na^+^ from soil exchange sites and assisted with restoring ionic homeostasis in the plant [76]. Also, foliar spraying of α-Toco does enhance the nutritional status of the plants, but not through the supply of nutrients directly: they act instead by helping fortify certain physiological and biochemical functions [25]. These findings were similar to the results of Lalarukh et al. [79] and Ali et al. [60] on sunflower. The main function of α-Toco is to prevent cellular membranes, especially plasma membranes, chloroplasts, and mitochondria, from oxidative damage through the quenching of ROS [22]. This is a critical dual action: protecting chloroplasts and mitochondria guarantees the continuous generation of the energy (ATP) and reducing power (NADPH) required to drive metabolism [80, 81]. Therefore, under stressful conditions, the plant can vigorously drive active transport and utilization of major nutrients such as N, P, and K, while it can reinforce selective uptake to limit Na^+^ entry and enhance its extrusion for better ionic homeostasis [82].

Biochar-induced changes in metabolite levels represent a metabolic reprogramming, which switched the plant’s strategy from survival to production. The amelioration in soil physicochemical properties relieved the abiotic stress to a great extent, and the evidence was the reduction of free proline [83]. Stress, therefore, was relieved from the plants so that they could allocate all the resources normally assigned to osmotic adjustment for the accumulation of TSS [78]. This increased activity of the primary metabolism is very likely to be directly related to an improved nutritional status of the plant, as the previously reported (Table 6) strong increase in leaf N and Mg supplies the material needed for a higher photosynthetic capacity. Likewise, the increased N absorption was also critical in enhancing the shikimic acid pathway. Since nothing is lacking in terms of carbon skeleton (increased TSS) and nitrogenous precursors, important enzymes as phenylalanine ammonia-lyase (PAL) are induced [84]. The high increase in antioxidative compounds implies that the biochar could have activated certain genetic routes that lead to a primed state of defense for the plant [85]. The accumulation of TPC, TFC, and EAT directly contributed to the lower IC₅₀ values recorded (Table 7), as these metabolites act as powerful hydrogen donors due to their hydroxyl-rich chemical structure [86]. Unlike the effects of biochar on soil, the foliar application of α-Toco promoted phytochemical synthesis by direct physiological action [22]. This direct action provides a double-layered protection: it retains the stability and efficiency of the photosynthetic apparatus and thus an adequate carbon skeleton (TSS) supply, and it maintains a functional status of several principal biosynthetic enzymes of the phenylpropanoid route [70]. In addition, α-Toco has been found to serve as a signaling molecule, upregulating the defense genes of plants themselves. This possibly accounts for the remarkable elevation in the EAT, establishing a positive feedback loop that strengthens the antioxidative system of plants. The overall effect is a biochemical profile enhanced in TPC and TFC. This is explained by the chemical structure containing hydroxyl groups; these metabolites act as powerful hydrogen donors [87], thus correlating with the substantially lower IC₅₀ values and the added overall antioxidant capacity recorded in this study.

The application of biochar meaningfully reinforced the plant’s defense mechanism in terms of enzymes against oxidative stress, which concurred with the results reported on the positive influence of biochar in different plants, such as mint [84], American ginseng [78], and common thyme [88]. Reduction in the levels of H₂O₂ and MDA observed is not simply a manifestation of an improved growth rate but is part of an avoidance strategy mediated by the ameliorated rhizosphere [83]. Through the restriction of toxic ion absorption and the enhancement of water potential, biochar attenuates the early oxidative burst in chloroplasts and mitochondria and thus stabilizes membranes [65]. At the same time, biochar treatment functions as a bio-stimulator, to prime defense system of the plant with expression of stress-related genes. The up-regulation in SOD and APX activities (Table 8) suggests a biochar-mediated priming effect, which enhanced the plant’s capacity to detoxify ROS, directly shielding cellular lipids and reducing MDA levels [85, 86]. In addition to the priming effect of biochar, the foliar spray of α-Toco acted as an important second line of defense via a dual-capacity mechanism. Mainly, as a strong lipophilic antioxidant, it directly penetrates through cell membranes and nuclei and terminates the lipid peroxidation chain reaction [80]. This scavenging process results in membrane stability, and this stabilizing effect is responsible for the dramatic decrease in MDA contents. Beyond structural support, α-Toco enhanced SOD and APX activities [75], likely by protecting chloroplast integrity and minimizing the oxidative inactivation of these enzymes under salinity stress [89].

Biochar application substantially enhanced the agronomic performance of milk thistle, which includes significantly earlier flowering and a marked increase in all yield components. In principle, this enhancement stems from an improvement in the health of the plant, which is corroborated by the increased uptake of essential nutrients (Table 6) and decreased levels of oxidative stress markers (Table 8). The significant reduction in number of days to 50% flowering suggests that by reducing edaphic stress conditions, biochar also enabled the plant to reallocate metabolic resources from stress defense to development, and in so doing to speed up its phenological progress through the vegetative stage, eventually producing a transition to reproduction at an earlier age [90]. The subsequent increases in the number of capitula per plant, capitulum diameter, and seeds per capitulum also result from the enhanced vigor. The accumulation of key macronutrients, specifically P and K, through biochar application is pivotal in more reproductive physiology [91]. Phosphorus is essential for energy transfer (ATP) during flower initiation and seed development, and potassium is important in the movement of photoassimilates from the leaves (source) to the developing capitula (sink) [19]. This enhanced source of carbohydrates (TSS, Table 7) provided energy for accomplishing these events [92]. Importantly, the increase in yield was not just in terms of quantity but also in quality. The improvement in flavonolignan and total silymarin content of seeds is a direct consequence of these same physiological improvements [91]. The greater availability of precursors due to increased TSS and N uptake along with upregulation of plant secondary metabolic roles—in this case due to the surplus energy not spent on stress responses—would create the ideal conditions toward increased silymarin biosynthesis [84]. Consequently, the significant increment in seed yield per plant, along with a higher 1000-seed weight and superior silymarin content, at least indicates that the plant was producing more and better-quality seeds, which ultimately translates to a maximized yield of a medicinally superior product per hectare [93]. Likewise, the foliar feeding of α-Toco also exhibited strong physiological biostimulant effects, promoting reproductive development and all the yield constituents. This acceleration was conferred by the protective action of α-Toco on photosynthetic apparatus against oxidative damage (Table 8). It ensures production of metabolic energy (ATP/NADPH) by preserving chloroplast integrity and delaying leaf senescence till the time the energy becomes critical in shifting to the reproductive phase as well as prolonging the period important for seed filling [80]. This raised metabolism was essential for yield formation. The steady flow of photo-assimilates from a functioning source (leaves) directly promoted the growth of a stronger sink (capitula), achieving remarkable increases in the number of capitula, seeds per capitulum, and individual seed weight [25, 78]. Importantly, the energy and carbon precursor surplus (TSS, Table 7) was channelled also towards the phenylpropanoid pathway, leading to the production of taxifolin and coniferyl alcohol, which are major moieties required for silymarin production [94]. Hence, the action of α-Toco was dual: it stimulated the yield of quantitative seed per plant, as well as the qualitative (medicinal) contribution of each seed. This also ensures the maximum yield of total bioactive silymarin per hectare; this therefore supports the use of this approach as a means of producing a more powerful and valuable final product.

The structural changes in anatomy described in Tables 11 and 12 are the ultimate structural confirmations for the previous physiological and yield enhancements. The influence of biochar, via its edaphic action, on the internal transport system of plant was direct, indicated by the remarkable enlargement of the vascular system—the thickening of the midvein bundles accompanied by an increase in the width of the XVD [95]. Structural development here provides the physical basis for enhanced hydraulic conductance and thus nutrient translocation, benefiting the up-regulated nutrient uptake (Table 6) and whole plant biomass. In parallel, the foliar spray of α-Toco, which promoted the formation of more efficient photosynthetic tissues, the enhanced maturation of which was due to increased LBT caused by the expansion of PTT and STT, favored mitigation of oxidative stress because of sheer increased metabolic activity [81], with elevation in TSS and more robust phytochemical synthesis (Table 7). Finally, these anatomical changes put together reflect the physical manifestation of the success of the treatments as they form the structural basis of the observed increases in both seed quantity and medicinal quality.

This research presents a powerful agronomic strategy derived from the positive synergism between biochar and α-Toco for growing medicinal plants under abiotic stress. One important element of our results is that the effective combination of these treatments is consistent with current sustainable agriculture, providing a dual-action approach. Whereas biochar is a long-lasting edaphic conditioner, with potential to enhance physical and chemical properties of soil to relieve stresses, α-Toco is a physiological bio-stimulant with focal action that boosts the endogenous antioxidant system and the secondary metabolism of the plant. From a commercial perspective, the integrated approach is highly feasible. Biochar is a sustainable soil amendment, and α-Toco is active at low dose, their joint use is therefore economical in case of large-scale application. Hence, their proven potential to synergistically enhance both seed yield and bioactive silymarin content holds obvious economic ramifications for the therapeutic-pharmaceutical sector, by enabling a high-value raw material that meets both agricultural scalability and industrial pharmaceutical quality. Additionally, from an economic point of view, the use of local biochar (derived from mango waste, at a modest cost of US$ 50 t⁻¹) combined with α-Toco was highly economically viable and profitable. Considering the maximum seed yield of 1.95 t ha⁻¹, which compares favorably against recent agronomic reports (e.g., [96, 97]), and a safe estimate for the farm-gate price of US$ 2,500 t⁻¹ [98, 99], it was found that the treatment with 10 t ha⁻¹ of biochar to milk thistle plants grown under salt stress was the most economically viable with a net profit of US$ 4,295 ha^− 1^, deducting a total variable cost of US$ 580 ha^− 1^ which comprised US 80 for application of α-Toco spray. This result reflects a high Benefit-Cost Ratio (BCR) of 8.40, indicating that for every dollar invested in this combined strategy, a return of US 4,045 ha⁻¹ due to a higher input cost of US$ 830 ha⁻¹, thus demonstrating the clear trade-off between maximizing the phytochemical quality and achieving the maximum economic return. In addition to the economic context of this combined strategy, environmental sustainability is also a factor of serious concern. Although biochar is a useful method of carbon sequestration, its mass production requires the strict filtering of clean feedstocks, including the mango residues of the area used in the present investigation, to reduce the possibility of trace metal pollution in the farm soils. At the same time, exogenous application of α-Toco is an eco-friendly intervention; being a natural, entirely biodegradable antioxidant, it leaves no critical residual effects to the ecosystem, thus ensuring that the intervention is safe and sustainable when it comes to saline soil restoration. Despite these promising agronomic, economic, and environmental outcomes, it is important to acknowledge the methodological limitations of the current study. This experiment was conducted under specific arid climatic conditions in Fayoum, Egypt, focusing on a single medicinal plant species and specific application rates. Furthermore, the long-term field stability of the applied biochar and its prolonged interactions with soil microbiota over multiple growing seasons were not evaluated. Therefore, future large-scale, multi-location field trials involving diverse soil types, varying environmental conditions, and different crop species are required to fully validate these findings and establish generalized global recommendations (Table 13).

**Table 13:** Economic analysis and cost-benefit assumptions for the biochar and α-tocopherol treatments

Treatment	10-t ha⁻¹ biochar + α-Toco	15-t ha⁻¹ biochar + α-Toco	Source/Ref.
Seed Yield t ha⁻¹	1.95	1.95	Present study
Farm-gate Price (US$ t⁻¹)	2,500	2,500	[98, 99]
Gross Return (US$ ha⁻¹)	4,875	4,875	Calculated
Total Variable Cost (US$ ha⁻¹)	580	830	Local market
Net Profit (US$ ha⁻¹)	4,295	4,045	Calculated
Benefit-Cost Ratio (BCR)	8.40	5.87	Calculated

## Conclusion

Salinization, which is brought about and aggravated by global climate change, is an increasing threat to sustaining agriculture. In this light, the present study introduces a unique, environment-friendly approach to support the productivity of milk thistle in salt-affected areas by synergistically applying biochar and α-Toco. Indeed, the results demonstrate that this approach effectively improved salinity tolerance by enhancing all growth parameters, photosynthetic performance, and physiological status. On the mechanistic side, this was achieved by restoring key leaf nutrient homeostasis, enhancing essential nutrient uptake, and significantly reducing toxic Na⁺ accumulation. At a biochemical level, the treatment induced a defensive metabolic shift by upregulating antioxidant capacity, reducing oxidative stress markers, and stimulating antioxidant enzyme activities. These combined effects, accompanied by positive anatomical adaptations, finally culminated in better phenological development, superior yield components, and the highest silymarin productivity. Consequently, on the basis of the economic feasibility analysis, the application of 10 t ha^− 1^ biochar plus 150 ppm α-Toco is proposed as the most profitable commercial solution. This combination offers a highly profitable net monetary return (US$ 4,295 ha^− 1^) while sustaining maximum seed yield (1.95 t ha^− 1^), thus providing a profitable and sustainable option to farmers working in salt-affected areas. Future research should focus on the long-term effects of these amendments on soil microbiome dynamics and the molecular mechanisms underlying this stress tolerance.

## Data Availability

All data generated or analyzed during this study are included in this published article.
